# The gut microbiome variability of a butterflyfish increases on severely degraded Caribbean reefs

**DOI:** 10.1038/s42003-022-03679-0

**Published:** 2022-07-30

**Authors:** Friederike Clever, Jade M. Sourisse, Richard F. Preziosi, Jonathan A. Eisen, E. Catalina Rodriguez Guerra, Jarrod J. Scott, Laetitia G. E. Wilkins, Andrew H. Altieri, W. Owen McMillan, Matthieu Leray

**Affiliations:** 1 Smithsonian Tropical Research Institute, Apartado, 0843-03092 Balboa, Ancon Republic of Panama; 2 Department of Natural Sciences, Manchester Metropolitan University, Manchester, M1 5GD UK; 3 Swire Institute of Marine Science; School of Biological Sciences, The University of Hong Kong, Pok Fu Lam Road, Hong Kong SAR, China; 4 School of Biological and Marine Sciences, University of Plymouth, Plymouth, PL4 8AA UK; 5 Genome and Biomedical Sciences Facility, University of California, Davis, 451 Health Science Drive, Davis, CA 95616 USA; 6 Department of Evolution and Ecology, University of California, Davis, Davis, CA 95616 USA; 7 Department of Medical Microbiology and Immunology, University of California, Davis, Davis, CA 95616 USA; 8 Max Planck Research Group Eco-Evolutionary Interactions, Max Planck Institute for Marine Microbiology, Celsiusstrasse 1, 28209 Bremen, Germany; 9 Department of Environmental Engineering Sciences, University of Florida, Gainesville, FL 32611 USA

**Keywords:** Metagenomics, Biodiversity, Microbiome, Microbial ecology

## Abstract

Environmental degradation has the potential to alter key mutualisms that underlie the structure and function of ecological communities. How microbial communities associated with fishes vary across populations and in relation to habitat characteristics remains largely unknown despite their fundamental roles in host nutrition and immunity. We find significant differences in the gut microbiome composition of a facultative coral-feeding butterflyfish (*Chaetodon capistratus*) across Caribbean reefs that differ markedly in live coral cover (∼0–30%). Fish gut microbiomes were significantly more variable at degraded reefs, a pattern driven by changes in the relative abundance of the most common taxa potentially associated with stress. We also demonstrate that fish gut microbiomes on severely degraded reefs have a lower abundance of *Endozoicomonas* and a higher diversity of anaerobic fermentative bacteria, which may suggest a less coral dominated diet. The observed shifts in fish gut bacterial communities across the habitat gradient extend to a small set of potentially beneficial host associated bacteria (i.e., the core microbiome) suggesting essential fish-microbiome interactions may be vulnerable to severe coral degradation.

## Abstract (web summary)

The gut microbiome composition of the coral-feeding butterflyfish across Caribbean reefs is more variable at degraded reefs. These microbiomes have a lower abundance of Endozoicomonas and a higher diversity of anaerobic fermentative bacteria.

## Introduction

Environmental degradation associated with the Anthropocene is threatening the persistence of mutualistic relationships that are key to the stability of ecological functioning^1^. The increasingly severe degradation of coral reefs from both local and climatic stressors has led to novel habitat states with conspicuously altered fish and invertebrate communities^2–5^, making them a model system for studying ecological responses to environmental change. A potentially pervasive but largely overlooked response to habitat degradation is the change to host-associated microbiomes—the communities of bacteria, archaea, fungi, unicellular eukaryotes, protozoa, and viruses that live on internal and external surfaces of reef organisms. Host microbiomes potentially respond faster than their hosts to changing environmental conditions and can promote acclimatization processes as well as genetic adaptation^6–10^. Thus, microbial communities could play a key role in mediating a host’s resilience and ability to adapt to environmental change. However, it remains to be explored whether mutualisms between fish hosts and gut microbiomes can shift to alternative beneficial relationships to provide a mechanism of resilience to habitat change, or whether the mutualism breaks down and simply reflects a cascading effect of degradation at all levels of ecological organization.

The importance of gut microbial communities in maintaining host health is well recognized in mammals and other vertebrates^11,12^, including a wealth of research into the importance of microbes in fish in aquaculture settings^13–16^. Fish harbor microbiomes that are unique from the microbial communities in their surrounding environment^17,18^. As the gut microbiome diversifies throughout the development of the fish host, a relatively stable gut microbiome is typically established within the first months of the fish’s life^13^. These resident (autochthonous) microbes, which are consistently found associated with the fish population across space and time and potentially provide critical functions for the host, are referred to as the “core microbiome”^15,19,20^. In contrast, the numerous microbes occurring in the gastrointestinal tract after being ingested are transient (allochthonous) and may vary intraspecifically with the developmental stage and potentially include opportunistic pathogens. Because of their importance in maintaining host metabolic homeostasis, the degree of stability of the core microbiome across a range of environmental conditions emerges as a key trait for predicting the resilience of host populations in aquatic animals^21–23^.

In coral reef fishes, recent studies have suggested that intestinal microbiomes influence key physiological functions associated with nutrient acquisition, metabolic homeostasis, and immunity^24–27^. For example, gut bacteria provide many herbivorous fish hosts with the ability to digest complex algal polymers^24,26,27^ and appear susceptible to human disturbances such as eutrophication^28^. The gut microbiome is also a major factor in the innate immune responses to a wide variety of pathogenic microorganisms and other stressors in the surrounding environment^29,30^. Given the rapid physical, chemical, and biotic changes affecting coral reefs, especially in the light of increasing mass coral bleaching events^31^, it is essential to gain a better understanding of how fish gut microbiome assemblages respond to environmental variation so that we can assess how these mutualisms govern host health and resilience to habitat change.

Here, we examined the variability and composition of the gut microbiome of the facultative coral-feeding foureye butterflyfish, *Chaetodon capistratus* (Linnaeus, 1758), inhabiting a set of reefs that differ markedly in coral cover and diversity across a tropical coastal lagoon (Bahía Almirante) at Bocas del Toro on the Caribbean coast of Panamá. The Chaetodontidae family (butterflyfishes) is among the largest and most iconic families of coral reef-associated fishes^32^ and an ideal group for studying microbiome responses to habitat degradation. Chaetodontids range from extreme diet specialists to facultative corallivores and generalists capable of consuming different types of prey such as corals, algae, polychaetes or crustaceans^33–35^. Due to their intimate link to the reef benthos, specialized coral-feeding species of Indo-Pacific butterflyfishes are highly sensitive to reductions in coral cover^36–38^. *Chaetodon capistratus* is the only one of the four Western Atlantic *Chaetodon* species with a relatively high proportion of anthozoans in its diet (mainly hard and soft corals)^39–41^. Because of this relative specialization, we chose it as a model species to study relationships between reef habitats and fish host gut microbiomes.

The Bahía Almirante encompasses an inner bay of protected reefs subjected to seasonally high temperatures and a watershed delivering nutrients from agriculture and sewage. In 2010, the bay faced an unprecedented hypoxic event, which led to massive coral bleaching and mortality on some sheltered reefs while others located near the bay’s mouth remained unaffected^42^. We capitalized on this gradient of habitat states across the bay to detect variation across fish gut microbiomes in relation to coral degradation. We hypothesized that fish residing on more degraded reefs (i.e., low live coral cover) have a more diverse and variable microbiome as a result of alternative feeding behaviors and potentially increased stress^43^. In contrast, given its role in sustaining host biological functions, we expected that the core microbiome would remain consistent across the habitat gradient.

## Results

### Benthic habitat and fish density

**Fig. 1:**
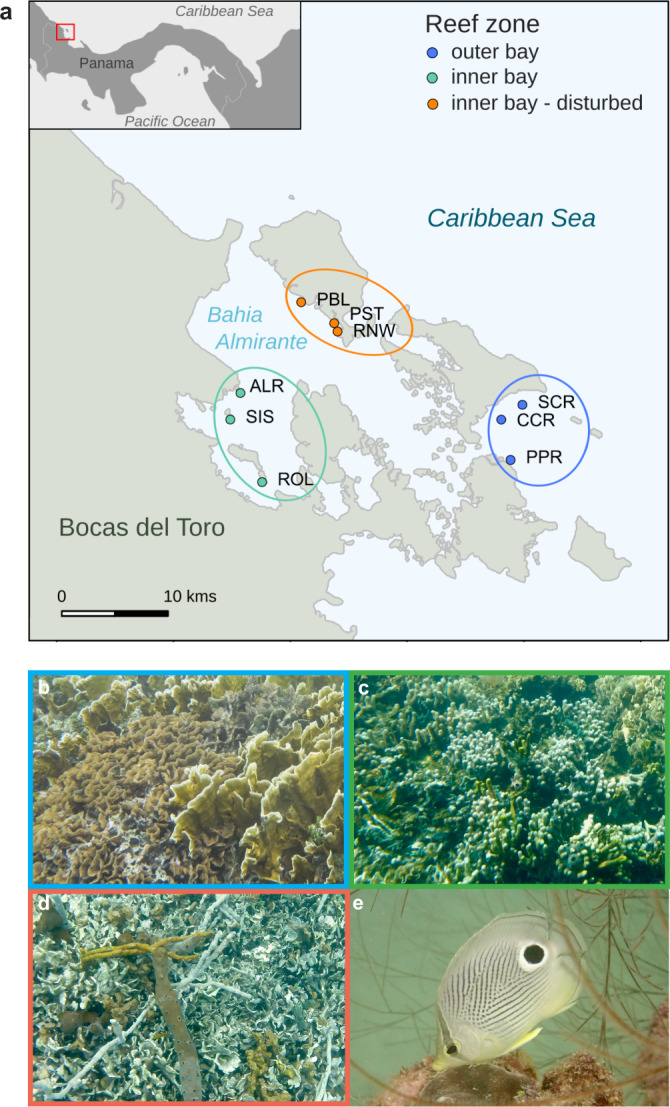
Study area and fish species. **a** Map of the Bahía Almirante (Bocas del Toro, Panamá) indicating the position of the nine reefs where samples were collected (generated using GSHHG version 2.3.7 https://www.soest.hawaii.edu/pwessel/gshhg/). Data: Friederike Clever. **b** Outer bay reefs with highest levels of live coral cover, **c** inner bay reefs with intermediate levels of coral cover, and **d** reefs located in the inner bay disturbed zone were highly impacted by a hypoxic event in 2010. **e** The study species foureye butterflyfish (*Chaetodon capistratus*). Photographs by Matthieu Leray.

**Fig. 2:**
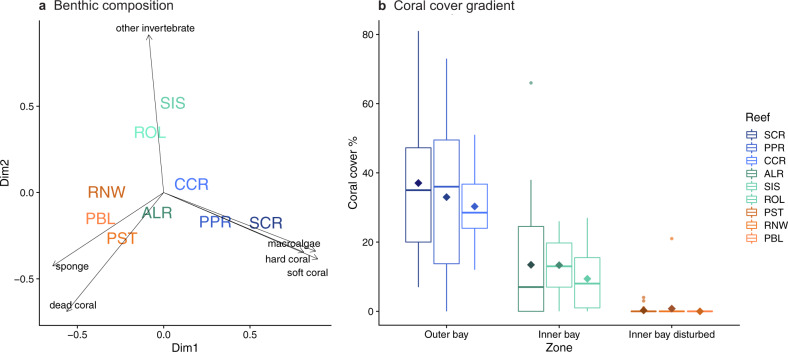
Benthic communities. Composition and percent coral cover of benthic communities across nine reefs and three reef zones illustrating a habitat gradient: **a** PCoA representing dissimilarities in benthic community composition based on Bray–Curtis. Reefs are color-coded by reef zone, substrate groups are depicted in black; **b** percent live coral cover across reef zones from high coral cover at the outer bay to very low cover on disturbed reefs at the inner bay. Diamond shapes depict means.

Reefs located within the three zones classified a priori as outer bay, inner bay, and inner bay disturbed (Fig. 1a), differed in terms of their benthic composition (PCoA; Fig. 2a) with marked differences in the level of live coral cover (Fig. 2b and Table S1). Live hard coral cover (Fig. 2b and Table S1) and coral diversity (Shannon diversity; Fig. S1) were highest on reefs of the outer bay. Both stony coral species (i.e., *Acropora cervicornis* and *Agaricia tenuifolia*) and fire corals (i.e., *Millepora alcicornis, Millepora complanata*) dominated at outer bay reefs. In the inner bay zone, reefs displayed an intermediate level of live coral cover (Fig. 2b and Table S1), largely dominated by the lettuce coral *Agaricia tenuifolia*. Sponges represented more than a quarter of the benthic cover on these reefs (Fig. S2 and Table S1). Live coral cover was lowest in the inner bay disturbed zone (Fig. 2b) where dead coral skeleton was prevalent together with sponges (Fig. S2 and Table S1). Our focal species *Chaetodon capistratus* showed significantly lower mean density levels at the outer bay than in the two inner bay zones (Fig. S3). Density levels were similar (1–5 individuals per 100 m^2^ transect) across all surveyed reefs inside of the bay apart from Cayo Hermanas (SIS, inner bay zone) where up to 25 individuals were recorded in one of the transects (Fig. S3).

### Composition of the whole gut microbiome

A total of 5,245,987 high-quality reads were retained for subsequent statistical analyses. The number of reads per sample ranged from 10,369 to 79,466, with a mean ± SD of 41,307 ± 10,990 reads. We identified 10,711 different ASVs in the total dataset. The number of ASVs per sample ranged from 13 to 1,281, with a mean ± SD of 179 ± 210 ASVs. This dataset primarily comprised ASVs belonging to 15 bacterial phyla (Fig. S4a). As predicted, *C. capistratus’* gut microbiome composition was distinct from the microbiome in seawater and the microbiome of potential prey items (sessile invertebrates) (Fig. S4a, b). *Chaetodon capistratus’* overall gut microbiome was dominated by Proteobacteria (mainly Gammaproteobacteria, 68.6%) followed by Firmicutes (16.1%), Spirochetes (9.27%), Cyanobacteria (3.98%) (Fig. S4a). Bacteria in the phylum Proteobacteria (Alpha-, Delta-, and Gammaproteobacteria) were dominated by a single genus (*Endozoicomonas*) in the gut of *C. capistratus* (93.9%) (Fig. S4b). Endozoicomonas were also abundant in hard- and soft coral samples (23.36 and 41.25% respectively. Firmicutes was abundant in fish guts (16.1% of fish gut bacteria) but representatives of this phylum were nearly absent from potential prey and seawater (Fig. S4a, b). Venn diagrams revealed that fish gut microbiomes shared largely similar proportions of ASVs with coral and sponge microbiomes in each zone (Fig. S5a–c). Fish shared a slightly higher proportion of ASVs with corals (hard and soft coral microbiomes combined) in the inner bay (6.6%; Fig. S5b) and inner bay disturbed zones (6.38%; Fig. S5c) than at the outer bay (5.43%; Fig. S5a). The proportion of shared ASVs between fish gut- and sponge microbiomes was lowest at the inner bay (2.58%; Fig. S5b) and highest at the inner bay disturbed zone (3.41%; Fig. S5c).

### Composition of the core gut microbiome

Indicator analysis identified 27 ASVs in eight families (i.e., Endozoicomonadaceae, Brevinemataceae, Ruminococcaceae, Lachnospiraceae, Vibrionaceae, Peptostreptococcaceae, Clostridiaceae, Thermaceae) as part of the “core” microbiome associated with the fish intestinal tract (Fig. S6 and Table S2). The genus *Endozoicomonas* (phylum Proteobacteria, class Gammaproteobacteria), described as a symbiont of marine invertebrates^44^, comprised 71.3% of the ASVs in the core followed by genus *Brevinema* (phylum Spirochetes, class Spirochaetia) (10.7%) and anaerobic fermentative bacteria in the families Ruminococcaceae (9.7%), Lachnospiraceae (5.6%), and Clostridiaceae (1.7%) (phylum Firmicutes, class Clostridia) (Fig. S6).

Blastn searches against nr/nt NCBI database revealed that ASVs identified as part of the core gut microbiome (i.e., *Endozoicomonas*) were previously found in scleractinian and soft coral tissue at our study area and in Curaçao among other locations (Table 1). Some *Endozoicomonas* ASVs were closely related to sequences identified previously in sponges, clams, ascidians, tunicates, and coral mucus as well as the intestinal tract of a coral reef fish species (*Pomacanthus sexstriatus*). Sequences assigned to Ruminococcaceae closely resembled bacteria reported from herbivorous marine fishes (*Kyphosus sydneyanus, Naso tonganus, Acanthurus nigrofuscus,* and *Siganus canaliculatus*), the omnivorous coral reef fish *Pomacanthus sexstriatus* and a freshwater fish. An *Epulopiscium* ASV matched to a sequence detected in the guts of two coral reef fishes, the omnivore *Naso tonganus* and the carnivore *Lutjanus bohar* and to sequences found in the coral *Orbicella faveolata*. Other Lachnospiraceae bacteria found in this study resembled sequences known from cattle rumen, hot springs, farm waste, human and other animal feces. Within Ruminococcaceae in Firmicutes, ASVs assigned to the genus *Flavonifractor* closely resembled bacteria reported from the hindgut of the temperate herbivorous marine fish *Kyphosus sydneyanus* in New Zealand. *Brevinema* sequences similar to ours have been previously isolated from the gut of the coral reef fish *Naso tonganus* as well as freshwater and intertidal fish intestinal tracts. Retrieved Vibrionaceae (genus *Vibrio*) were similar to sequences found in a coral reef fish gut of *Zebrasoma desjardinii*. A *Romboutsia* ASV (family Peptostreptococcaceae), a recently described genus of anaerobic, fermentative bacteria associated with the intestinal tract of animals including humans^45–47^, which also occurs in mangrove sediments^48^, matched a sequence found in the tissue of the sea fan *Gorgonia ventalina* at our study site Bocas del Toro (Table 1).

**Table 1:** Basic local alignment search tool for nucleotides (BLASTn)^119^ search results for ASVs identified as part of the core microbiome to infer where these ASVs or close sequences have been previously identified.

ASV ID	Taxon	% Identity	Isolation source	Host group	Host species	Country	Ocean/River	Reference
ASV1	Endozoicomonas	100	coral tissue	scleractinian coral	*Porites astreoides*	Panama (Bocas del Toro)	Western Atlantic	Sunagawa 2010^77^
		100	coral tissue	scleractinian coral	*Orbicella faveolata*	Panama (Bocas del Toro)	Western Atlantic	Sunagawa et al. 2009^73^
		100	coral tissue	scleractinian coral	*Orbicella annularis*	Curaçao	Western Atlantic	Klaus et al. 2007^136^
ASV5	Endozoicomonas	99.6	GI tract	coral reef fish	*Pomacanthus sexstriatus*	NP	NP	Ward et al. 2009^137^
		99.21	coral tissue	scleractinian coral	*Porites astreoides*	Panama (Bocas del Toro)	Western Atlantic	Sunagawa et al. 2010^77^
ASV6	Endozoicomonas	100	coral tissue	scleractinian coral	*Porites astreoides*	Panama (Bocas del Toro)	Western Atlantic	Sunagawa et al. 2010^77^
ASV11	Endozoicomonas	99.6	coral tissue	scleractinian coral	*Porites lutea*	South Africa	Western Indian Ocean	Sere et al. 2013^138^
		99.6	coral tissue	scleractinian coral	NP	Thailand, Ko Tao	Western South China Sea	Roder et al. 2014^74^
		99.6	coral tissue	scleractinian coral	*Porites* sp.	Panama (Bocas del Toro)	Western Atlantic	Roder 2014^74^
ASV9	Flavonifractor	98.2	GI tract	marine fish	*Kyphosus sydneyanus*	New Zealand	South-Western Pacific	Moran et al. 2005^139^
ASV14	Ruminococcaceae	98.42	GI tract	coral reef fish	*Naso tonganus*	Australia (Great Barrier Reef)	Pacific	Mendell et al. 2010 Accession: HM630215
ASV7	Endozoicomonas	98.81	gill	bivalve mollusc (clam)	*Ctena orbiculata*	Florida, Sugarloaf Key	Western Atlantic	Lim et al. 2017 Accession: KY687505
		98.81	gill	bivalve mollusc (clam)	*Loripes lacteus*	Meditarranean	Meditarranean	Mausz et al. 2008^140^
		98.81	sponge tissue	sponge	*Theonella swinhoei*	China	South China Sea	Feng 2015 Accession: KT121420
ASV2	Brevinema	93.7	GI tract	coral reef fish	*Naso tonganus*	Australia (Great Barrier Reef)	Pacific	Mendell et al. 2010 Accession: HM630215
		93.68	GI tract	marine and brakish fish	*Gillichthys mirabilis*	United States (California)	Pacific	Bano et al. 2007^141^
ASV3	Endozoicomonas	100	coral mucus	scleractinian coral	NP	Curaçao	Western Atlantic	Frade et al. 2016^142^
		100	coral tissue	scleractinian coral	*Porites astreoides*	Panama (Bocas del Toro)	Western Atlantic	Sunagawa et al. 2010^77^
ASV17	Endozoicomonas	99.6	coral tissue	scleractinian coral	*Porites astreoides*	Panama (Bocas del Toro)	Western Atlantic	Sunagawa et al. 2010^77^
		99.6	coral mucus	scleractinian coral	NP	Curacao	Western Atlantic	Frade et al. 2016^142^
ASV18	Ruminococcaceae	95.26	GI tract	freshwater fish	*Thymallus* sp.	Russia	Bol’shaya Tira River	Sukhanova et al. 2011 Accession:HE584732
		95.28	biogas reactor	reactor water	NP	Japan (Hokkaido)	NP	Nishioka et al. 2019 Accession: LC473933
ASV10	Lachnospiraceae	94.7	rumen	black beef cattle	NP	Japan	NP	Koike 2013 Accession:AB821803
		94.7	feces	human	*Homo sapiens*	NP	NP	Turnbaugh et al. 2009^143^
		94.7	feces	human	*Homo sapiens*	United States	NP	Ley et al. 2006^144^
ASV27	Epulopiscium	100	coral tissue	scleractinian coral	*Orbicella faveolata*	Puerto Rico	Western Atlantic	Kimes et al. 2013^145^
		100	GI tract	coral reef fish	*Naso tonganus*	Australia (Great Barrier Reef)	Pacific	Mendell et al. 2010 Accession:HM630230
		100	GI tract (distal intestine, feces)	coral reef fish	*Lutjanus bohar*	Palmyra Atoll	Pacific	Smriga et al. 2010^146^
ASV15	Ruminococcaceae	98.41	GI tract	coral reef fish	*Acanthurus nigrofuscus*	Saudi Arabia	Red Sea	Miyake et al. 2015^26^
		98.02	GI tract	coral reef fish	*Siganus canaliculatus*	China	NP	Zhang et al. 2018^147^
		96.43	feces	kangaroo	*Macropus rufus*	Australia	NP	Ley et al. 2008^11^
ASV68	Endozoicomonas	99,60	tissue	tunicates	NP	Malaysia	Western South China Sea	Danish-Daniel et al. 2018 Accession:MG896199
		99,60	tissue	ascidian	*Styela clava*	Denmark	NP	Schreiber et al. 2016 Accession: KU648381
		99,60	coral tissue	scleractinian coral	*Colpophyllia natans*	Curacao	NP	Klaus et al. 2011^148^
ASV30	Romboutsia	100	soft coral tissue	soft coral	*Gorgonia ventalina*	Panama (Bocas del Toro)	Western Atlantic	Sunagawa et al. 2010^77^
ASV95	Vibrio	99,61	GI tract	coral reef fish	*Zebrasoma desjardinii*	Saudi Arabia	Red Sea	Miyake et al. 2016^68^
		99,60	water	water	NP	Brazil	NP	Coutinho et al. 2012 Accession: JQ480694
		99,21	marine sediment	marine sediment	NP	India (Andaman Islands)	Indian Ocean	Cherian et al. 2019 Accession: MK975459
ASV94	Romboutsia	NP	NP	NP	NP	NP	NP	NP
ASV19	Clostridium sensu stricto 1	99.6	feces	goose	*Branta canadensis*	Canada	NA	Lu et al. 2009^149^
		99.21	aquaponic biofilm	NP	NA	Mexico	NA	Munguia-Fragozo et al. 2016 Accession: KY125439
		98.81	feces	human child	*Homo sapiens*	Nigeria	NP	Tidjani Alou et al. 2016 Accession: LT161894
ASV24	Tyzzerella	97.23	suspended plant residue in a methanogenic reactor of cattle farm waste	NP	NP	NP	NA	Ueki et al. 2017^150^
ASV25	Ruminococcaceae	98.02	fish gut	coral reef fish	*Acanthurus nigrofuscus*	Saudi Arabia	Red Sea	Miyake et al. 2016^68^
		97.62	fish gut	coral reef fish	*Siganus canaliculatus*	China	South China Sea	Juan et al. Accession: HG970996
		96.03	feces	red kangaroo	*Macropus rufus*	USA, Saint Louis Zoological Park	NA	Ley et al. 2008^11^
		95.28	GI tract	coral reef fish	*Pomacanthus sexstriatus*	NP	NP	Ward et al. Accession:EU885024
ASV39	Anaerofilum	97.62	fish gut	coral reef fish	*Acanthurus nigrofuscus*	Saudi Arabia	Red Sea	Miyake et al. 2016^68^
		97.22	fish gut	coral reef fish	*Siganus canaliculatus*	China	South China Sea	Juan et al. Accession: HG970996
		96.83	GI tract	coral reef fish	*Naso tonganus*	Australia (Great Barrier Reef)	Pacific	Mendell et al. Accession: HM630257
ASV41	Epulopiscium	100	coral mucus	scleractinian coral	NP	Curacao	Western Atlantic	Frade et al. 2016^142^
		100	freshwater microbialite	NA	NA	Mexico	NP	Corman et al. Accession:KP479649
ASV59	Endozoicomonas	99.21	bivalve gill	bivalve mollusc (clam)	*Ctena orbiculata*	USA, Florida	Atlantic	Lim et al. Accession: KY687505,
		99.21	pharynx tissue	ascidian	*Ascidia* sp.	Sweden	North Sea	Schreiber et al. Accession: KU64822
		99.21	gill	bivalve mollusc (clam)	*Loripes lacteus*	NP	Mediterranean	Mausz et al. Accession: GQ853556
ASV74	Clostridium sensu stricto 2	98.02	contaminated groundwater	NA	NA	USA	NA	Bowman et al. 2008^151^
ASV163	Clostridium sensu stricto 2	100	tunicates	tunicate	NP	Malaysia	NP	Danish-Daniel et al. ACCESSION: MG896199
		100	pharynx tissue	ascidian	*Ascidia* sp.	Sweden	North Sea	Schreiber et al. ACCESSION: KU648273
ASV589	Thermus	100	plant root	plant	NP	USA	NA	Bueno de Mesquita et al 2020^152^

Core ASVs were compared to the non-redundant nucleotide (nr/nt) collection database of the National Centre for Biotechnology Information (NCBI) with BLASTn. Metadata are recorded for sequences that matched each query at 100% similarity or the first five top hits. *NP* information not provided, *NA* not applicable.

### Alpha diversity of the whole gut microbiome

**Fig. 3:**
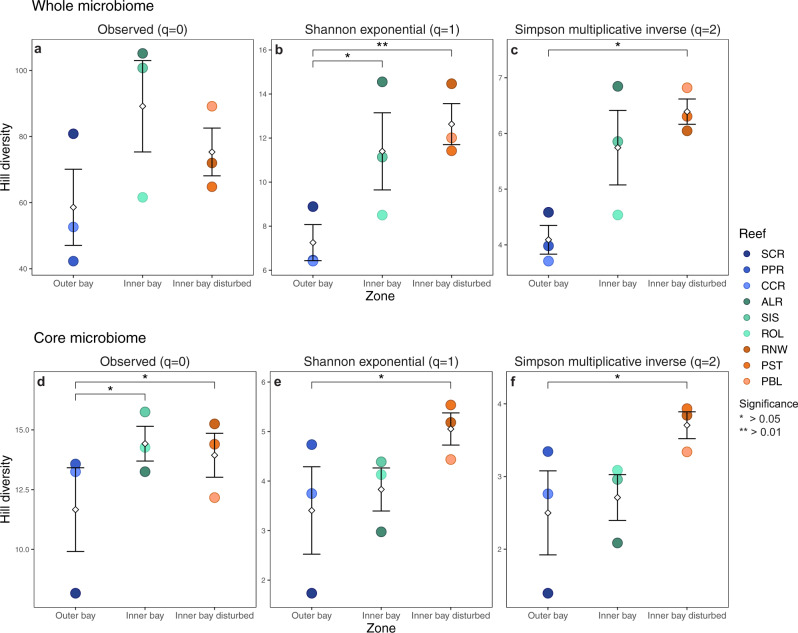
Alpha diversity. Differences in diversity (mean ± SE) of ASVs between the whole gut microbiome (**a**–**c**) and the core gut microbiome (**d**–**f**) of *Chaetodon capistratus* across reefs. Alpha diversity was measured based on Hill numbers using three metrics that put more or less weight on common species. The observed richness (**a**, **d**) does not take into account relative abundances. Shannon exponential (**b**, **e**) weighs ASVs by their frequency. Simpson multiplicative inverse (**c**, **f**) overweighs abundant ASVs. Significance depicts differences in alpha diversity among reef zones (Kruskal–Wallis test with post hoc Dunn test). Diamonds depict means.

We estimated alpha diversity using Hill numbers of three different orders of diversity (Hill numbers, {q = 0, 1, 2}) that place more or less weight on the relative abundance of ASVs. This approach allowed for balancing the representation of rare ASVs that might be the result of sequencing errors. Diversity of the whole gut microbiome was lower in fish of the outer bay zone than in fish of the inner bay and inner bay disturbed zones (Fig. 3a–c). Diversity differed significantly among the three zones when taking into account ASV frequency with the Shannon index (Fig. 3b) and when emphasizing abundant ASVs with the Simpson index (Fig. 3c and Table S3). However, observed ASV richness did not significantly differ among zones (Fig. 3a and Table S3). Benjamin–Hochberg corrected post hoc tests showed significantly higher Shannon diversity in fish guts of the inner bay zone versus the outer bay zone (Table S4). Fish of the inner bay disturbed zone had a higher microbial diversity than fish of the outer bay zone based on both Shannon and Simpson (Table S4). Pairwise comparisons of alpha diversity between reefs revealed that fish that resided on the reef with the highest level of coral cover overall (37.07%), Salt Creek (SCR, outer bay), had a significantly lower diversity of microbes in their guts than fish from all three inner bay disturbed reefs (RNW, PST, and PBL) for both Shannon and Simpson diversity (Table S5).

### Alpha diversity of the core gut microbiome

Diversity of ASVs in the core microbiome was lowest at the outer bay when comparing ASV richness among fish of the outer bay, inner bay, and inner bay disturbed zones and was highest in fish in the inner bay disturbed zone with both the Shannon index and Simpson index (Fig. 3d–f). Alpha diversity differed significantly among the three zones (Table S3) and pairwise testing revealed that this was largely due to differences between fish of the outer bay and inner bay disturbed zones (Table S4). When compared by reef, lower core microbial diversity in fish from Salt Creek (SCR, outer bay) than fish from other reefs across all zones was responsible for the most significant comparisons (Table S5).

### Beta diversity of the whole gut microbiome

**Fig. 4:**
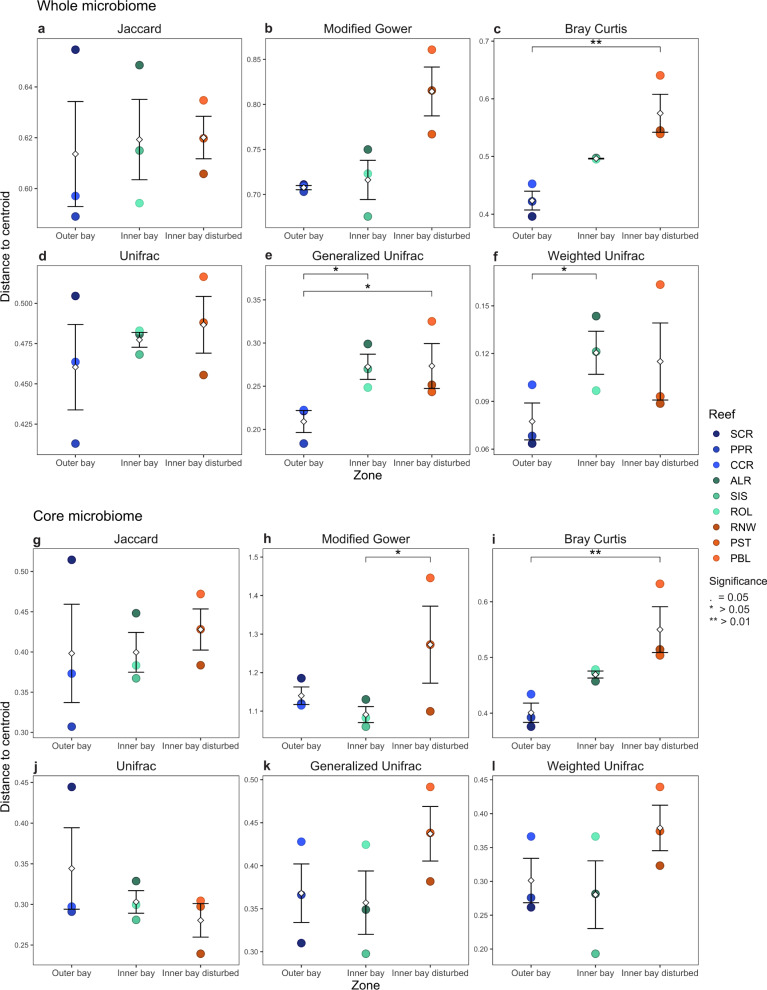
Multivariate dispersion. Compositional variability of the whole gut microbiome (**a**–**f**) and core gut microbiome (**g**–**l**) of *Chaetodon capistratus* across reefs. Compositional variability is measured as the distance to the centroid (mean ± SE) of each group (fish at each reef) in multivariate space. Multivariate analyses were computed with non-phylogenetic (Jaccard: panels **a**, **g**; Modified Gower: panels **b**, **h**; and Bray–Curtis: panels **c**, **i**) and phylogenetic (Unifrac: panels **d**, **j**; Generalized Unifrac: **e**, **k**; Weighted Unifrac **f**, **l**) metrics that differ in how much weight they give to relative abundances. On one end of the spectrum, Jaccard and Unifrac only use presence-absence data, whereas on the other end of the spectrum Bray–Curtis and Weighted Unifrac give a lot of weight to abundant ASVs in dissimilarity calculations. Significance depicts differences in multivariate dispersion between reef zones (ANOVA). Diamonds depict means.

Permutational Analysis of Multivariate Dispersion (PERMDISP2) indicated no difference in variability in the whole fish gut microbiome across zones and reefs using dissimilarity metrics that put limited weight on abundant ASVs (Fig. 4a, b and Table S6). However, Bray–Curtis, which more heavily weighs abundant ASVs, identified significantly higher multivariate dispersion for fish from the inner bay disturbed zone than for fish from the outer bay zone (Fig. 4c and Table S6). The same pattern was observed with phylogenetic dissimilarity metrics. Only the two metrics taking into account relative abundances (i.e., GUniFrac and WUniFrac) revealed significant differences in dispersion patterns among the three zones. Using GUniFrac, an index that adjusts the weight of abundant ASVs based on tree branch lengths, gut microbiomes of fish from the inner bay disturbed zone were significantly more spread in multivariate space than gut microbiomes of fish from the outer bay zone (Fig. 4e and Table S6). Gut microbial communities were significantly more variable in fish from the inner bay zone than in fish from the outer bay zone using both GUniFrac and WUniFrac (Fig. 4e, f and Table S6).

**Fig. 5:**
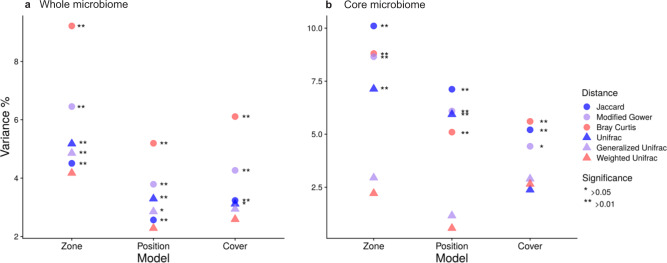
PERMANOVA. Proportion of the variance explained in Permutational Analysis of Variance (PERMANOVA) comparing the composition of the whole gut microbiome (**a**) and the core gut microbiome (**b**) of *Chaetodon capistratus*. Three independent PERMANOVA analyses were conducted. The “zone” model compares gut microbiomes among the three zones of the bay (inner bay, inner bay disturbed, and outer bay). The “position” model contrasts the composition of gut microbiomes of fish collected at reefs inside and outside of the bay. The “cover” model compares gut microbiomes of fish on disturbed and undisturbed reefs inside of the bay. Three non-phylogenetic (circles) and three phylogenetic (triangles) dissimilarity metrics were used. They place more (red) or less (blue) weight on relative abundances.

The three PERMANOVA models explained a small portion of the variance in the composition of the whole gut microbiome using all metrics (2.29–9.22%; Fig. 5a and Table S8). Nevertheless, gut microbiome composition was significantly different between fish from all three zones (zone model), between fish collected inside and outside the bay (position model) and between fish collected on inner bay reefs that differ in coral cover (cover model) when using Jaccard, modified Gower and Bray–Curtis distances (Fig. 5a and Table S8). Whole gut microbiomes differed using phylogenetic metrics UniFrac and GUniFrac but not when emphasizing microbial relative read abundance (WUniFrac) (Fig. 5a and Table S8). Pairwise Adonis with Bonferroni corrected *P* values revealed significant differences among all pairs of zones using non-phylogenetic metrics (Table S10). Pairwise tests were significant using the Unifrac distance except between gut microbiomes of fish from the inner bay and inner bay disturbed zones. None of the pairwise tests using GUnifrac and WUnifrac were significantly different among zones (Table S10).

Whole fish gut microbiomes featured differential relative read abundances across reefs of the inner bay disturbed, inner bay, and outer bay zones (Fig. S7). Gut microbiomes of fish from the inner bay disturbed zone had a lower proportion of microbial reads assigned to Endozoicomonadaceae (Proteobacteria), but a higher proportion of Vibrionaceae and Rhodobacteraceae. In contrast, the relative contribution of Spirochetes and Firmicutes was highest in the guts of fish in the inner bay disturbed zone (Fig. S7). Within Spirochetes, the relative abundance of Brevinemataceae was highest in gut microbiomes of fish from the inner bay disturbed zone, while Clostrideaceae within Firmicutes contributed more to gut microbiomes of fish on inner bay reefs but relatively little to the gut microbiomes of fish in the outer bay zone. Shewanellaceae (phylum Proteobacteria) represented a higher proportion of the gut microbiome of fish on inner bay disturbed reefs (Fig. S7).

### Beta diversity of the core gut microbiome

Patterns in multivariate dispersion were largely consistent between whole and core gut microbiomes. Differences among the three reef zones were significant for metrics that place more weight on ASV relative abundance (common ASVs) (Fig. 4h, i and Table S7). The variability of the core gut microbiome differed significantly between fish from the inner bay and inner bay disturbed zones and between fish from the inner bay disturbed and outer bay zones with highest variability levels in the inner bay disturbed zone. However, none of the phylogenetic metrics showed significant differences in dispersion among zones (Fig. 4j–l and Table S7)

As with the whole gut microbiome, the three PERMANOVA models explained a limited amount of variance in the composition of the core gut microbiome (Fig. 5b and Table S9). Yet, composition differed significantly among fish from the three zones (zone model), between fish in the inner bay and outer bay zones (position model) as well as between zones of differential coral cover within the bay (cover model) (Fig. 5b and Table S9). The core gut microbiome appeared largely similar in composition using all phylogenetic metrics but Unifrac (Table S9). Similar to the whole microbiome, pairwise Adonis tests with Bonferroni corrected *P* values showed significant differences among almost all pairs of zones when using taxonomic metrics (Table S11). Of the phylogenetic metrics, the only significant differences were found between the inner versus outer bay, and between the inner bay disturbed versus outer bay zone using Unifrac (Table S11). Differences in the composition of the core microbiome among reef zones was largely driven by changes in the relative abundance of ASVs assigned to the genus *Endozoicomonas* (class Gammaproteobacteria) (Fig. S5b). For example, the most common *Endozoicomonas* ASV (ASV1) was much more represented in the guts of fish from the outer bay and inner bay zones than in the guts of fish in the inner bay disturbed zone, while *Endozoicomonas* ASVs relative abundances appeared more evenly distributed towards the inner bay disturbed zone. In contrast, bacteria in the genus *Brevinema* (phylum Spirochetes) were most abundant relative to other members of the core in fish of the inner bay disturbed zone and least abundant in the outer bay zones. The giant bacterium *Epulopiscium* (family Lachnospiraceae, order Clostridia), which is known to aid the digestion of algae in surgeonfishes, contributed more to the core gut microbiome of fish on reefs in the inner bay disturbed zone than the inner and outer bay zones (Fig. S5b).

### Prevalent ASVs in each reef zone

A machine learning-based, de-noising algorithm (PIME) was used to detect sets of ASVs in the whole gut microbiome that significantly contribute to differences between reef zones. The initial out-of-bag (OOB) error rate (i.e., the prediction error in a Random Forest model) for our unfiltered dataset was greater than 0.1 (PIME, OOB 0.27) indicating that PIME filtering would effectively remove noise. PIME identified a prevalence cut-off of 65% for the highest improved accuracy (OOB = 2.25) indicating that the model was 97.75% accurate (Table S12). The validation step showed that randomized errors (Fig. S8b) corresponded with the predicted prevalence cut-off value of 0.65 indicating the absence of false positives (Type I error).

**Fig. 6:**
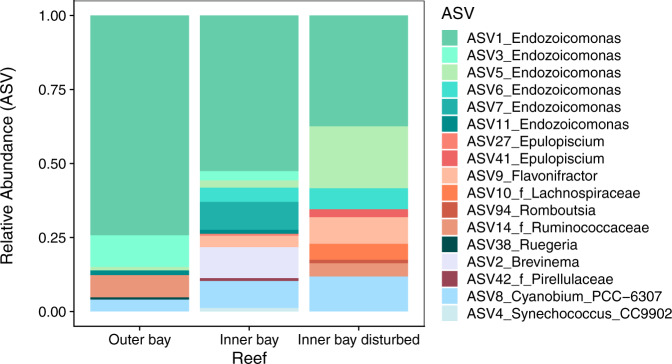
PIME filtering zones 65% prevalence. Comparison of fish gut microbiomes among three reef zones. The whole fish gut microbial dataset was filtered using Prevalence Interval for Microbiome Evaluation (PIME)^134^ to detect which ASVs were responsible for differences among zones. Using machine learning, PIME de-noises the data by reducing within-group variability. Based on the algorithm, we selected a 65% prevalence cut-off resulting in a filtered dataset of 17 ASVs at a low error rate (OOB = 2.25) and high model accuracy (97.75%).

After selecting ASVs that were present in at least 65% of the fish guts within each zone, the filtered dataset comprised 17 ASVs in eight families; i.e., Endozoicomonadaceae, Ruminococcaceae, Pirellulaceae, Lachnospiraceae, Brevinemataceae, Cyanobiaceae, Rhodobacteraceae, and Peptostreptococcaceae (Fig. 6 and Tables S12, S13,S14). Fish of the inner bay zone showed the highest richness levels with 13 ASVs, compared to eight and nine ASVs in fish of outer bay and inner bay disturbed zones, respectively (Fig. 6). An *Endozoicomonas* ASV (ASV1), which was also a dominant component of the core, had a much higher relative abundance in fish of the outer bay zone than in fish of the inner bay disturbed zone (Fig. 6). Communities differed most in composition between fish of the outer bay and inner bay disturbed zone, whereas fish of the inner bay zone reflected an intermediate community between these two comprising the highest richness of *Endozoicomonas* with six ASVs. As in the core community, the *Endozoicomonas* assemblage was slightly less diverse in the disturbed zone (three ASVs) and featured more similar relative read abundances than in the outer bay zone (four ASVs) where a single ASV was dominant. Two distinct ASVs of the giant bacterium *Epulopiscium* (family Lachnospiraceae) were prevalent in fish in both the inner and inner bay disturbed zones but were more abundant on disturbed reefs. Disturbed reefs uniquely featured anaerobic gut bacteria in the genus *Romboutsia* (family Peptostreptococcaceae) (Fig. 6).

## Discussion

Detecting how the spatial turnover of microbiomes varies within and among host populations, and in relation to habitat characteristics is essential to understanding and predicting the response of host species to environmental change. We show that whole gut microbial communities were significantly more diverse and variable in fish from inner bay disturbed reefs than from the outer bay zone in terms of some but not all measured diversity components. Conspicuously, the core microbiome, a small set of microbial strains that may form sustained relationships with the fish host, also showed higher dispersion on degraded reefs suggesting greater variability of microbial assemblages among individual fish. Significant differences in diversity and group dispersion were observed mostly in the relative abundances of the frequent and common taxa. Highly variable host-associated microbial communities have been observed in humans with immunodeficiency syndromes (reviewed in ref. ^49^) and in marine animals such as scleractinian corals and anemones under acute stress^43,50–52^. Zaneveld et al.^43^ referred to this pattern of variability as the “Anna Karenina principle” applied to host-associated microbiomes (AKP). They argued that this is a common but often overlooked response of organisms that become unable to regulate their microbiome. Our results are consistent with patterns expected under the Anna Karenina principle, which could potentially imply that fish experience some level of stress in association with habitat degradation.

Reductions in coral cover may increase foraging costs if, for example, fish spent more energy to search, capture, and handle their prey. Indeed, physiological stress imposed by environmental conditions may cause immune signals that imbalance the gut microbiome^30,43,53,54^. Disturbance to the microbiome, in turn, can affect the brain and further alter behaviors related to movement such as the ability to forage^30,55^. The low coral cover may also increase stress through intra- and interspecific competition for resources. For example, social stress in the form of aggressive interactions among conspecifics was shown to alter the behavior and microbial assemblages associated with mice by setting off immune responses critical to host health^56^. In Indo-Pacific obligate feeding butterflyfishes coral degradation was shown to decrease aggressive encounters among and within *Chaetodon* species^57^ as well as change the frequency of pair formation^58^, and the way species responded to the loss of the coral resource depended on their level of dietary specialization^57,58^. Foraging on degraded reefs may also increase predation risk when architectural complexity is reduced^59^. Anxiety-like behaviors induced by exposure to predators can lead to sustained physiological stress in vertebrates (reviewed in ref. ^60^).

Another possible explanation for more variable gut microbiomes on disturbed reefs could be increased behavioral heterogeneity among fish individuals (e.g., feeding behavior). Where preferred food sources are scarce, foraging behavior may become more diverse and lead to increased individual specialization on alternative food items^61,62^ translating into more varied gut microbiomes. In this scenario, the higher variation in gut microbial assemblages would be the result of behavioral adjustments (acclimatization) to alternative habitat conditions without necessarily causing stress. Higher alpha diversity across fish gut microbiomes in the inner bay disturbed zone supports this explanation. Although *C. capistratus* is able to consume a broad range of diet items^39,63,64^, deviations from its preferred coral prey may come with fitness consequences as shown for Indo-Pacific butterflyfishes^33,65,66^. For example, other authors found that obligate corallivorous *Chaetodon* species have reduced energy reserves at reefs where they diversify or shift their diet in response to limited coral availability^33,66^.

Apart from patterns of microbiome variability, the significant differences in the composition of the whole gut microbiome (as opposed to the core microbiome) in nearly all comparisons (i.e., among all three zones, between inner and outer bay, and between inner bay disturbed and undisturbed) may primarily reflect changes in diet. Specifically, in the inner bay disturbed zone where coral cover was low, the microbial assemblage suggests (i) potential changes in the invertebrate prey community and (ii) a more broad, likely omnivorous trophic profile indicated by a distinct *Endozoicomonas* community in codominance with anaerobic fermentative bacteria. The increased prevalence of fermentative microbes at disturbed reefs might reflect the consumption of algae and potentially sponges given their high availability in this zone. However, we lack information on the effects of sponge consumption on fish gut microbiomes. *Epulopiscium*, often considered a host-specific symbiont of herbivorous surgeonfishes (family: Acanthuridae)^26,27,67,68^, was present in the core microbiome and identified as distinct to the inner bay with predominance at disturbed reefs. This may suggest that *C. capistratus* can assimilate nutrients from algae and that this metabolic function is enhanced on degraded reefs by the increase in key microbial functional groups. Alternatively, the fish in our study may take up these microbes while foraging for invertebrates on the epilithic algal matrix. Overall, levels of *Epulopiscium* were approximately similar to those previously found in omnivores and detritivores in the Red Sea^26^ with the two most prevalent ASVs matching a strain previously isolated from the turf algal grazer *Naso tonganus*^69^. Additionally, the presence of Rhodobacteraceae, which are often found associated with algal biofilms^70,71^, may suggest detritivory but might also be related to the consumption of mucus from stressed^72^ and diseased corals where Rhodobacteraceae are also found^73,74^. The lower relative abundance of a compositionally distinct *Endozoicomonas* community on disturbed reefs could reflect different proportions of prey species featuring *Endozoicomonas*^75,76^ in the diet of *C. capistratus*.

In contrast, a single dominant *Endozoicomonas* ASV along with a few Firmicutes characterized the gut microbiome of *C. capistratus* on outer bay reefs. The presence of some *Endozoicomonas* ASVs shared between fish guts and potential prey (i.e., hard corals, soft corals, zoanthids, sponges), including matches to microbial sequences previously detected in two coral species (*Orbicella faveolata* and *Poritis asteroides*) in our study area at Bocas del Toro^73,77^, suggests the horizontal acquisition of these microbes via feeding on corals. In addition, we identified an ASV in the genus *Ruegeria* as indicative of the outer bay reefs, which matched a sequence previously retrieved from the soft coral species *Pterogorgia anceps* on the Caribbean coast of Panamá (GenBank Accession: MG099582) and which was also present across samples of potential prey taxa including hard and soft corals and sponge-infauna. *Endozoicomonas* originating from the food could potentially play a role in promoting the assimilation of nutrients via interactions with resident bacteria^78^.

The core microbiome composition significantly differed across the inner bay between fish from disturbed and undisturbed reefs where environmental conditions are generally homogeneous except for the proportion of live coral cover. Despite our models accounting for relatively little variance, this finding may suggest that bacterial communities that are most likely to have intimate metabolic interactions with *C. capistratus* might fail to provide important functions to hosts in severely degraded habitats. However, we cannot exclude the contribution of other factors that were not measured in this study such as microbial plasticity mediated by diet, gut colonization history^79^, and/or potential genetic differentiation between the inner bay and outer bay fish populations^80–82^.

Our analysis identified ten *Endozoicomonas* ASVs as part of the core microbiome indicating potential true resident symbionts. Members of the genus *Endozoicomonas* spp. are known as bacterial symbionts of marine sessile and some mobile invertebrates and fishes^44,76,83–87^. Reverter et al. (2017)^86^ found *Endozoicomonas* associated with butterflyfish gill mucus in *Chaetodon lunulatus* and Parris et al. (2016)^87^ found *Endozoicomonas* in the gut of damselfishes (family: Pomacentridae) and cardinalfishes (family: Apogonidae) pre- and to a lesser extent post-settlement on the reef. Corallivory in butterflyfishes has evolved in close association with coral reefs^32,88^ and this likely involved adaptive mechanisms to metabolize defense compounds from corals and many other sessile invertebrates (e.g., polychaetes). Adapted gut microbial communities may help butterflyfish hosts cope with toxins or facilitate the digestion of complex prey tissues as in insects^89^, mammalian herbivores^90^, and surgeonfishes^26^. It is likely that the gut microbial profile of *C. capistratus* —featuring high abundance *Endozoicomonas*—facilitates the digestion of complex coral prey. More detailed knowledge will be required to understand whether the potential intake of *Endozoicomonas* via fish browsing on sessile invertebrates plays a role in nutrient uptake in trophic strategies such as fish corallivory.

We detected increases in gut microbiome variability, diversity, and spatial community turnover. These patterns extended to the core microbiome suggesting signs of potentially altered functioning that may affect fish hosts on reefs with extremely low levels of live coral cover. Nonetheless, the density of *C. capistratus* was comparable across both inner bay zones indicating that the lack of live coral cover may not immediately impact the persistence of populations. Significantly lower densities at the outer bay may potentially relate to spatial patterns of larval recruitment^91^ and/or differences in wave exposure across reefs affecting the energy expenditure fish allocate towards swimming performance and feeding^92^. Additional work should focus on linking changes in the gut microbiome to direct measures of diet and host health. Our results give insight into the poorly understood spatial fluctuations in host-associated microbial communities across a natural system. This work highlights intricate links between ecosystem-scale and microbial-scale processes, which have so far been mostly overlooked. We suggest there is an urgent need to integrate measurements of the role of microbes in the response of reef fishes to the global loss of coral reefs.

## Methods

### Study area

Bahía Almirante, located in the Bocas del Toro Archipelago on the Caribbean coast of Panamá, is a coastal lagoonal system of ~450 km^2^ where numerous, relatively small, and patchy fringing coral reefs occur^93^. Hydrographic and environmental conditions vary across the semi-enclosed bay but are generally characterized by limited water exchange with the open ocean^42^. Furthermore, areas of the bay are subjected to uncontrolled sewage and dredging due to increasing coastal development and agricultural runoffs from the adjacent mainland^94–97^. A total of nine discontiguous reefs distributed from the mouth towards the inner bay were selected for this study based on distinct hydro-geographical zones and disturbance history, resulting in three distinct reef zones with three replicates each (total *n* = 9 reefs) (Fig. 1a). Throughout the manuscript, we will refer to these three distinct reef zones as “outer bay”, “inner bay”, and “inner bay disturbed”. Outer bay reefs [Salt Creek (SCR), Cayo Corales (CCR), and Popa (PPR)] are located at the mouth of the bay marking a transition zone between the inner bay and the open ocean. These reefs represent typical Caribbean reef communities featuring both massive and branching coral colonies with higher benthic cover and diversity as compared to the inner bay (Fig. 1b). Inner bay reefs [Almirante (ALR), Cayo Hermanas (SIS), and Cayo Roldan (ROL)] are largely coral and sponge dominated reefs and have lower coral diversity than the outer bay reefs (Fig. 1c). Inner bay disturbed reefs [Punta Puebla (PBL), Punta STRI (PST) and Runway (RNW)] were heavily impacted by the 2010 hypoxic event^42^, which resulted in the current cover of largely dead coral comprised of formally prevalent *Agaricia* and *Porites* species (Fig. 1d). Prior to this disturbance, both study zones located inside of the bay exhibited comparable benthic communities of similar health states. For example, the Punta STRI reef (PST) at the now disturbed zone featured 26.9% coral cover in 2005^98^.

### Benthic habitat and fish density

Visual surveys of benthic cover and focal fish species densitiy were conducted between May and June 2016. At each of the nine reefs, three 20 m transects were placed parallel to the shore at 2–4 m depth. Benthic community cover was estimated from 100 cm × 70 cm photographic quadrats placed every 2 m, resulting in a total of 10 quadrats per transect. Photos were analyzed on the CoralNet platform^99^ using a stratified random sampling design (10 rows × 10 columns with 1 point per cell for a total of 100 points per image). The first 15 of all photos were manually scored to train the algorithm. The remaining photos were then processed by the automated assignment tool, and assignments were subsequently verified for each point. Due to the difficulty involved with photo-based taxonomic identification, analyses were conducted at the level of broad benthic categories which comprised the following: live hard coral, dead hard coral, live soft coral, sponge, zoanthid, other invertebrates, seagrass, grazable substrate, macroalgae, rubble, sand, shade and “unknown”. Within the live hard coral, dead hard coral, and live soft coral categories, identification was done at the genus or species level when possible. The mean cover of each benthic category was calculated per reef. To estimate focal fish species density, *C. capistratus* individuals were counted along each 20 × 5 m belt transects used subsequently for the benthic surveys while swimming slowly using scuba (except at CCR).

### Sample collection

The foureye butterflyfish, *Chaetodon capistratus*, is a common member of Caribbean coral reef fish communities (IUCN classified as least concern)^100^ with a distribution that extends across the subtropical Western Atlantic^101,102^ (Fig. 1e). The following protocol of fish capture and euthanization had been approved by the Smithsonian Tropical Research Institute’s Institutional Animal Care and Use Committee (IACUC). An average of 11 individual adult fish were collected at each of the nine reefs (min = 7; max = 16; total = 102) by spearfishing in February and March 2018 (Table S15). Captured fish were immediately brought to the boat, anesthetized with clove oil, and placed on ice in an individual and labeled sterile Whirl-Pak bag. Upon return to the research station, fish were weighed (g wet weight), and both standard length (mm SL) and total length (mm TL) were measured with a digital caliper. The intestinal tract of each fish was removed under a laminar flow hood using tools decontaminated with 10% sodium hypochlorite. The intestinal tracts were then preserved in 96% ethanol in individual 15 ml or 5 ml centrifuge tubes and stored at −20 °C until DNA extraction. To assess microbial communities present in the fish’s environment, we also obtained samples of seawater and potential prey taxa. At each of nine reefs, a total of four liters (2 × 2 L at each reef) of seawater was collected immediately above the reef substratum using sterile Whirl-Pak bags and filtered through a 0.22 µm nitrocellulose membrane (Millipore) and a total of 18 seawater samples was included into downstream analysis. Small pieces of hard coral (*Siderastrea siderea*, *n* = 2; *Porites furcata*, *n* = 2; *Agaricia tenuifolia n* = 2), soft coral (*Antillogorgia bipinnata*, *n* = 1; *Plexauridae* sp.; *n* = 1), sponges (*Amphimedon compressa*, *n* = 1; *Chondrilla caribensis*, *n* = 1; *Demospongiae* spp., *n* = 4), macroalgae (*Amphiroa* sp., *n* = 1), turf (*n* = 1), and zoantharia (*Zoanthus pulchellus, n* = 1; *Palythoa caribaoerum*, *n* = 1) were collected opportunistically at least at one of the three habitat zones and kept in sterile Whirl-Pak bags on ice on the boat. At the field station, samples were individually placed in 50 ml or 15 ml centrifuge tubes with 96% ethanol and stored at −20 °C until DNA extraction.

### DNA analysis

The mid- and hindgut of the gastrointestinal tract of each fish was opened longitudinally to isolate the digesta and the mucosa tissue by lightly scraping the intestinal epithelium. Between 0.05 and 0.25 g of both tissue types combined was used for DNA extraction using the Qiagen Powersoil DNA isolation kit following the manufacturer's instructions with minor modifications to increase yield (see supplementary methods). Each tissue sample of potential prey organisms (invertebrates and macroalgae) was homogenized in separate vials. Additionally, infaunal communities (small worms) were isolated from two sponges, *Amphimedon compressa* and *Dysidea* sp. and the tissue homogenized for each sponge separately. DNA was extracted (0.25 g per sample) following the same protocol as described for the intestinal microbiomes. Seawater DNA was isolated from nitrocellulose membrane filters using the Qiagen Powersoil Kit following a modified protocol described in ref. ^103^.

A dual Polymerase Chain Reaction (PCR) approach was used to amplify the V4 hypervariable region (primers 515F^104^ and 806R^105^) of the 16 S ribosomal rRNA gene of each sample (Table S16). Subsequently, the product of all samples was sequenced by combining them into a single Illumina MiSeq sequencing run. Our protocol followed the 16 S Illumina Amplicon Protocol of the Earth Microbiome Project^106^ using locus-specific primers to which Illumina “overhang” sequences were appended. These overhang sequences served as a template to add dual index Illumina sequencing adapters in a second PCR reaction (see supplementary methods for detailed PCR protocols). The final product was sequenced on the Illumina MiSeq sequencer (reagent kit version 2, 500 cycles) at the Smithsonian Tropical Research Institute with a 20% PhiX spike. The absence of contaminants was confirmed with negative DNA extractions and negative PCR amplifications (see supplementary methods for detailed DNA extraction and PCR protocols).

### Analysis of sequence data

All analyses were conducted with the statistical software R version 3.6.158^107^. Illumina adapter and primer sequences were removed from forward and reverse reads using Cutadapt^108^ with a maximum error rate of 0.12 (-e 0.12). The remaining reads were filtered and trimmed based on their quality profiles and potential chimeras were removed using DADA2 version 1.12.1^109^. Sequences were discarded if they had more than two expected errors (maxEE = 2), at least one ambiguous nucleotide (maxN = 0), or at least one base with a high probability of erroneous assignment (truncQ = 2). Forward and reverse reads were trimmed to 220 and 180 bp respectively to remove lower quality bases while maintaining sufficient overlap between paired-end reads. Sequences were kept when both the forward and reverse reads of a pair passed the filter. Quality filtered reads were de-replicated and Amplicon Sequence Variants (ASVs) inferred. Paired-end reads were merged and pairs of reads that did not match exactly were discarded. Taxonomy was assigned to each ASV using a DADA2 implementation of the naive Bayesian RDP classifier^110^ against the Silva reference database version 132^111^. ASVs identified as chloroplast, mitochondria, Eukaryota, or those that remained unidentified at the kingdom level were removed from the dataset. Sequences of each ASV were aligned using the DECIPHER package version 2.0^112^. The PHANGORN package version 2.5.5^113^ was then used to construct a maximum likelihood phylogenetic tree (GTR + G + I model) using a neighbor-joining tree as a starting point. Fourteen samples containing few sequences (<10,000) were removed from the dataset (Fig. S9). The remaining samples were rarified without replacement to even sequencing depth (*n* = 10,369 sequences) for downstream analysis. Our approach followed the recommendation for the normalization of sequencing data^114^. Statistical analysis was conducted using phyloseq version 1.28.0^115^.

### Delineation of the core gut microbiome

To identify the persistent bacteria associated with the fish gut (i.e., the “core microbiome”^19,116^) including taxa that might be potentially beneficial to the fish host, we employed a statistical approach taking into account both relative abundance and relative frequency of occurrence of ASVs as opposed to the common procedure of using an arbitrary minimum frequency threshold based on presence-absence data only^116^. Indicator species analysis^117^ (labdsv package version 2.0-1)^118^ was used to identify which ASVs were relatively more abundant and predominantly found in fish guts and not in their surrounding environment. We calculated an Indicator Value (IndVal) Index between each ASV and two groups of samples: (1) all fish gut samples, and (2) all seawater and sessile invertebrate samples upon which fish potentially feed. Statistical significance of the association between ASVs and groups of samples was tested using 1000 permutations. ASVs were considered indicators of fish guts (i.e., components of the core) based on a maximum probability of *P* value = 0.01. Sequences of ASVs identified as part of the core microbiome were compared to the non-redundant nucleotide (nr/nt) collection database of the National Centre for Biotechnology Information (NCBI) using the Basic Local Alignment Search Tool for nucleotides (BLASTn)^119^. We extracted metadata associated with all sequences that matched each query at 100% similarity or the first five top hits to identify within what taxa, environment, and/or habitat each core ASV and close relatives had been previously found.

### Diversity analysis

**Fig. 7:**
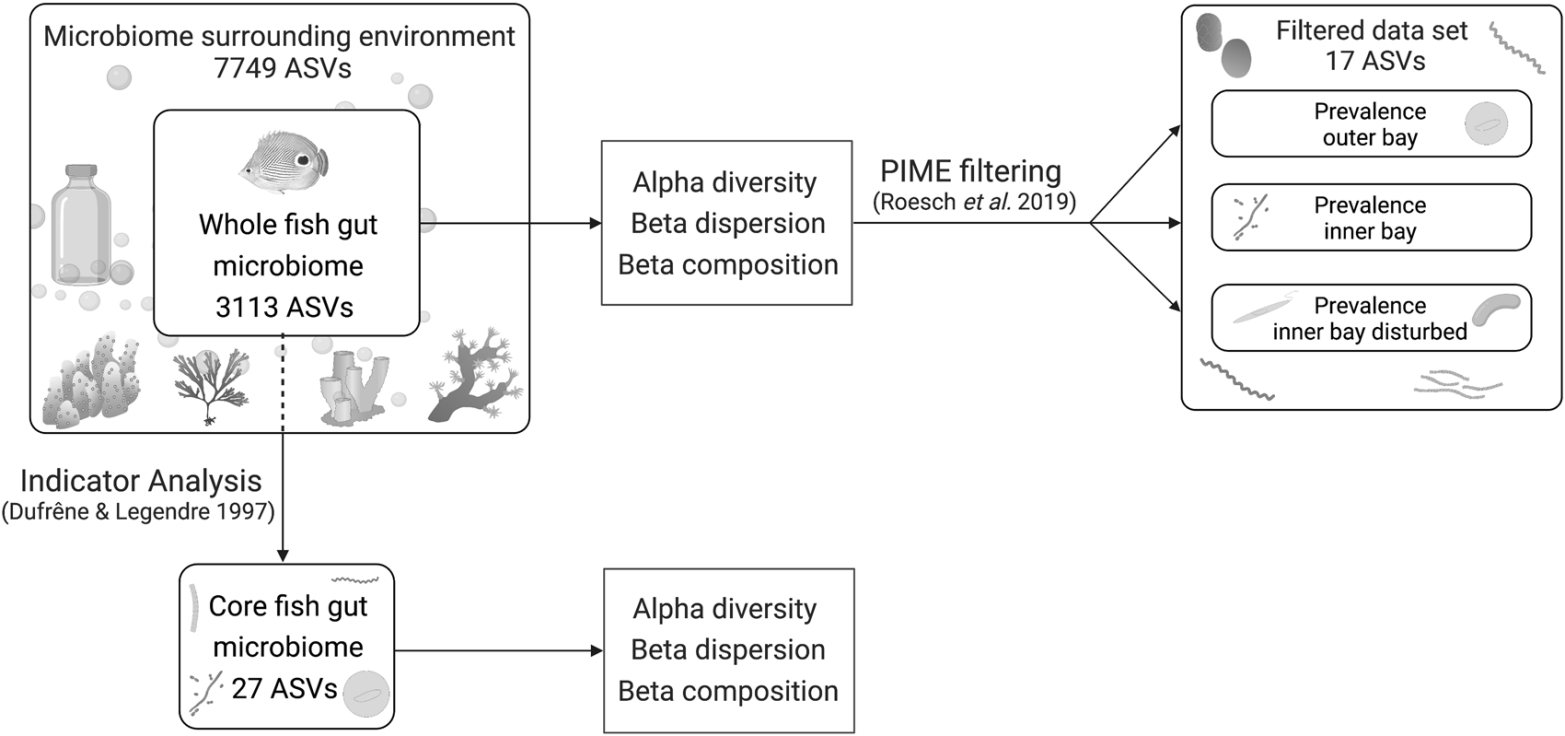
Microbial community analysis. Microbial community analysis workflow illustrating how we subsetted the whole fish gut microbiome dataset to delineate the core gut microbiome and gut microbial communities by zone, respectively. To identify the core microbiome, we used indicator analysis^117^ between the whole fish gut microbiome and the environmental sample fraction consisting of samples of potential fish prey taxa and the surrounding seawater. Diversity analysis was done for the whole and core fish gut microbiome, respectively. The whole fish gut microbiome was filtered for prevalence with a machine learning-based algorithm (PIME)^134^ to detect community differences among zones that reflect fish-microbiome responses to the habitat gradient. Created with BioRender.com. The fish icon is adapted from a color photograph of *Chaetodon capistratus* obtained from https://biogeodb.stri.si.edu/caribbean/en/pages with permission by D R Robertson. Icons of benthic organisms obtained from the IAN Symbol Libraries: Tracey Saxby and Joanna Woerner, Integration and Application Network (ian.umces.edu/media-library). https://creativecommons.org/licenses/by-sa/4.0/.

The workflow of our microbial community analysis is visualized in a diagram (Fig. 7). To account for the presence of rare sequence variants caused by sequencing errors or other technical artifacts^120^, we used Hill numbers^121^ following the approach recommended by ref. ^122^ for sequence data to compare alpha diversity among groups of samples. Hill numbers allow scaling the weight put on rare versus abundant sequence variants while providing intuitive comparisons of diversity levels using “effective number of ASVs” as a measuring unit^121–123^. This approach allowed for balancing the over-representation of rare ASVs that might be inflated due to sequencing errors^124^. We calculated three metrics that put more or less weight on common species: (1) observed richness, (2) Shannon exponential that weighs ASVs by their frequency, and (3) Simpson multiplicative inverse that overweighs abundant ASVs. Alpha diversity was calculated and visualized using boxplots for the whole and core fish gut microbiomes. Because Shapiro–Wilk tests indicated that the data were not normally distributed, non-parametric Kruskal–Wallis tests were used to compare alpha diversity among reefs (*n* = 9) and the three reef zones (outer bay, inner bay, and inner bay disturbed) with post hoc Dunn tests.

To test the hypothesis that fish gut microbiomes are more variable between individuals at disturbed sites, we calculated non-parametric Permutational Analysis of Multivariate Dispersion (PERMDISP2) (betadisper function, vegan package implemented in phyloseq^115,125^). PERMDISP2 is a measure of the homogeneity of variance among groups and compares the average within-group distance to centroid between each predefined group of samples in multidimensional space. We used a range of phylogenetic and non-phylogenetic dissimilarity metrics that differentially weigh the relative abundance of ASVs to identify the effect of abundant ASVs [Phylogenetic: Unifrac, Generalized Unifrac (package GUniFrac)^126^ and Weighted Unifrac^127^; non-phylogenetic: Jaccard^128^, modified Gower with log base 10^129^ and Bray–Curtis^130^]. *P* values were obtained by permuting model residuals of an ANOVA (Analysis of Variance) null-model 1000 times (betadisper function, vegan^115,125^).

Differences in microbial composition were tested using Permutational Multivariate Analysis of Variance (PERMANOVA) with the Adonis function in vegan^131^ computed with 10,000 permutations. Comparisons were made (1) among fish gut microbiomes of the three reef zones (“zone model”), (2) between fish gut microbiomes of outer bay reefs versus inner bay reefs (“position model”) and (3) between fish gut microbiomes of inner bay reefs and inner bay disturbed reefs which differed in coral cover (“cover model”). PERMANOVA is robust to the effect of heterogeneity of multivariate dispersions in balanced or near balanced designs as in our study^132^. Pairwise Adonis with Bonferroni corrected *P* values was computed using the pairwise Adonis function (version 0.4)^133^.

Finally, we used the Prevalence Interval for Microbiome Evaluation (PIME) package (version 0.1.0)^134^ to identify sets of ASVs that are frequently found in fish guts in each zone at the Bahía Almirante (outer bay, inner bay, inner bay disturbed). This analysis is aimed at identifying microbial ASVs that are differentially prevalent among zones. PIME uses a supervised machine learning Random Forest algorithm to reduce within-group variability by excluding low-frequency sequences potentially confounding community comparisons of microbiome data^134^. PIME identifies the best model to predict community differences between groups by defining a prevalence threshold that retains as many ASVs as possible in the resulting filtered communities (i.e., the random forest classifications) while minimizing prediction error (out-of-bag error, OOB). To do so, the algorithm uses bootstrap aggregating (100 iterations) of each sample group at each filtering step (prevalence interval) by 5% increments. Random Forest calculates a global prediction from a multitude of decision trees based on the bootstrap aggregations and estimates the out-of-bag error rate (OOB) from omitted subsamples during aggregating^135^. Validation was done by randomizing the original dataset (100 permutations) and subsequently estimating Random Forest error to determine if group differences in the filtered dataset were due to chance (pime.error.prediction function, PIME)^134^. A second function (pime.oob.replicate, PIME)^134^ repeated the Random Forest analysis using the filtered dataset for each prevalence interval without randomizing group identity. In a preliminary step, we assessed whether the OOB error for our unfiltered data was >0.1, which indicated that de-noising with PIME would improve model accuracy.

### Statistics and reproducibility

Statistical analyses were carried out for the 16 S sequencing data and in-situ transect data (benthic photographic quadrats and visual censuses of fish communities). Details allowing the reproducibility of all analyses are provided in the methods section (including sampling sizes and numbers of replicates). A diagram illustrating the statistical workflow is also included (Fig. 7) and the R code is available on the project website: https://github.com/bocasbiome/web/.

### Reporting summary

Further information on research design is available in the Nature Research Reporting Summary linked to this article.

## Supplementary information

Supplementary Information

Reporting Summary

## Data Availability

Sequencing data has been submitted to the NCBI Short Read Archive (SRA) database (https://www.ncbi.nlm.nih.gov/sra) under bioproject number Accession: PRJNA718434 ID: 718434. Raw data are available on Dryad Digital Repository 10.5061/dryad.m905qfv28^153^.

## Code availability

Source code is available at https://github.com/bocasbiome/web/^154^.
