# Supplementary material for: The gut microbiome variability of a butterflyfish increases on severely degraded Caribbean reefs
Source: Commun Biol. 2022 Jul 30;5:770. doi: 10.1038/s42003-022-03679-0 (PMC9338936; doi:10.1038/s42003-022-03679-0)
Supplement: Supplementary file 1 — Supplementary Information [file 42003_2022_3679_MOESM1_ESM.pdf]

Supplementary Information

The gut microbiome variability of a butterflyfish increases on severely degraded Caribbean reefs

Friederike Clever, Jade M. Sourisse, Richard F. Preziosi, Jonathan A. Eisen, E. Catalina Rodriguez Guerra, Jarrod J. Scott, Laetitia G.E. Wilkins, Andrew H. Altieri, W. Owen McMillan, Matthieu Leray

Index

Supplementary Figures 2
Supplementary Tables 11
Supplementary Methods 27
Supplementary References 28

I. Supplementary Figures

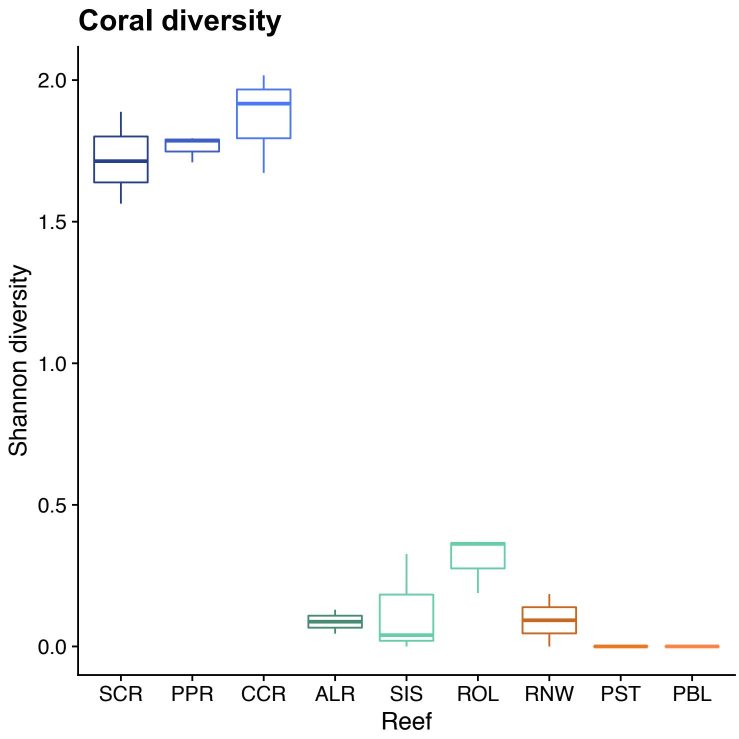

Figure S1. Shannon diversity of the coral community at each of nine reefs inferred from photographic quadrats along three transects per reef. The data reflect a gradient from high coral cover at the outer bay reefs (SCR, PPR, CCR) to the inner bay (ALR, SIS, ROL) and inner bay disturbed reefs (RNW, PST, PBL).

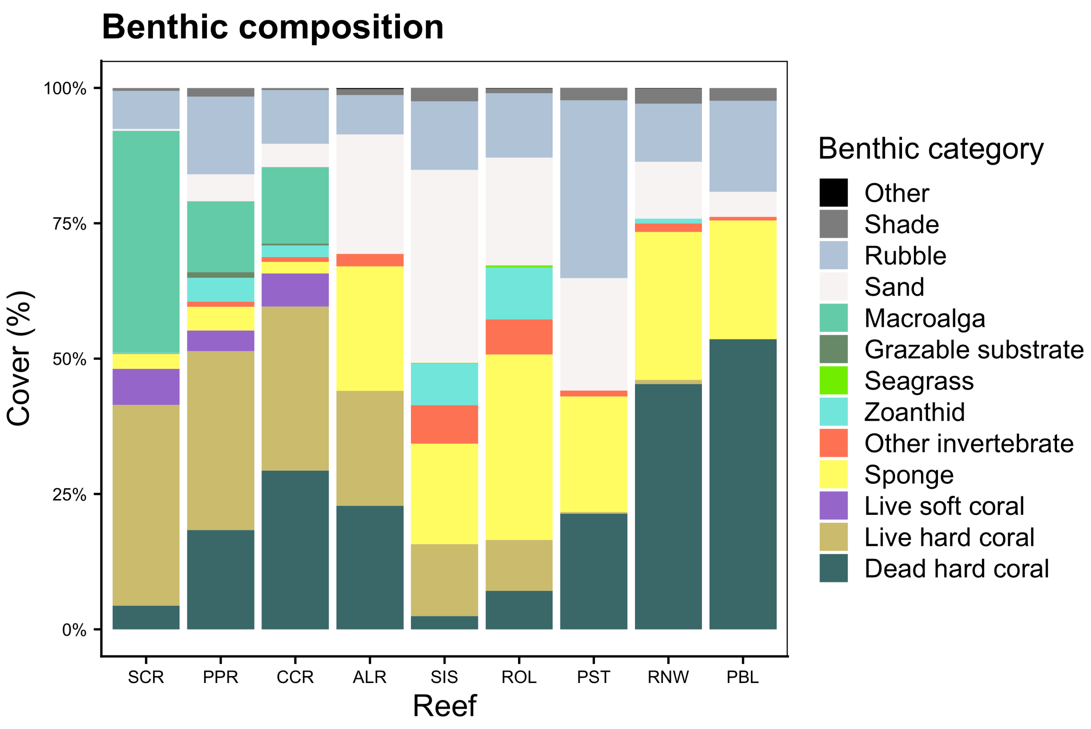

Figure S2 Percent cover of benthic species across nine study reefs based on data obtained from photo quadrats recorded along three 25 m transects per reef. Unidentified substrate was pooled in the "other" category.

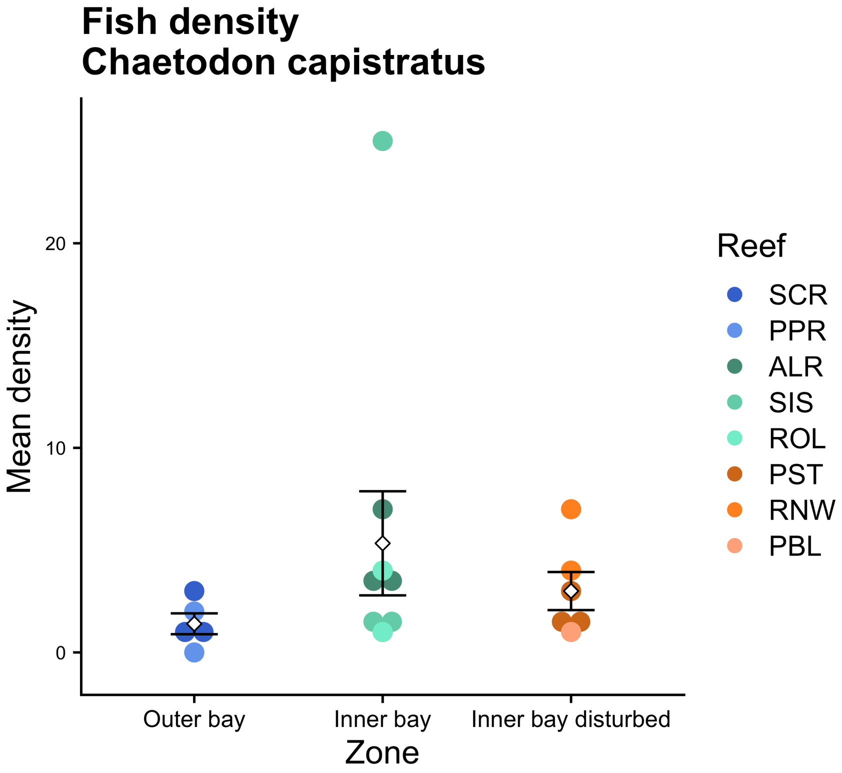

Figure S3 Study species (*Chaetodon capistratus*) mean densities across nine study reefs. Error bars represent standard errors. Reefs are colour-coded by reef zone: blue = outer bay, green = inner bay, orange = inner bay disturbed.

a **All sample fractions Phylum**

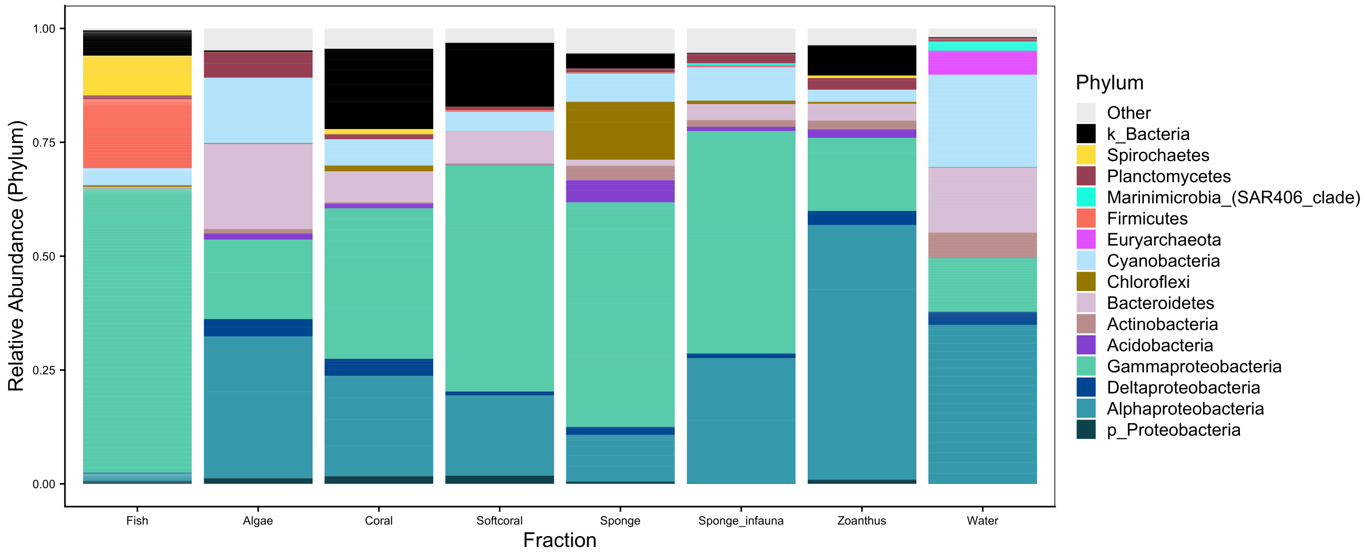

b **All sample fractions Genus**

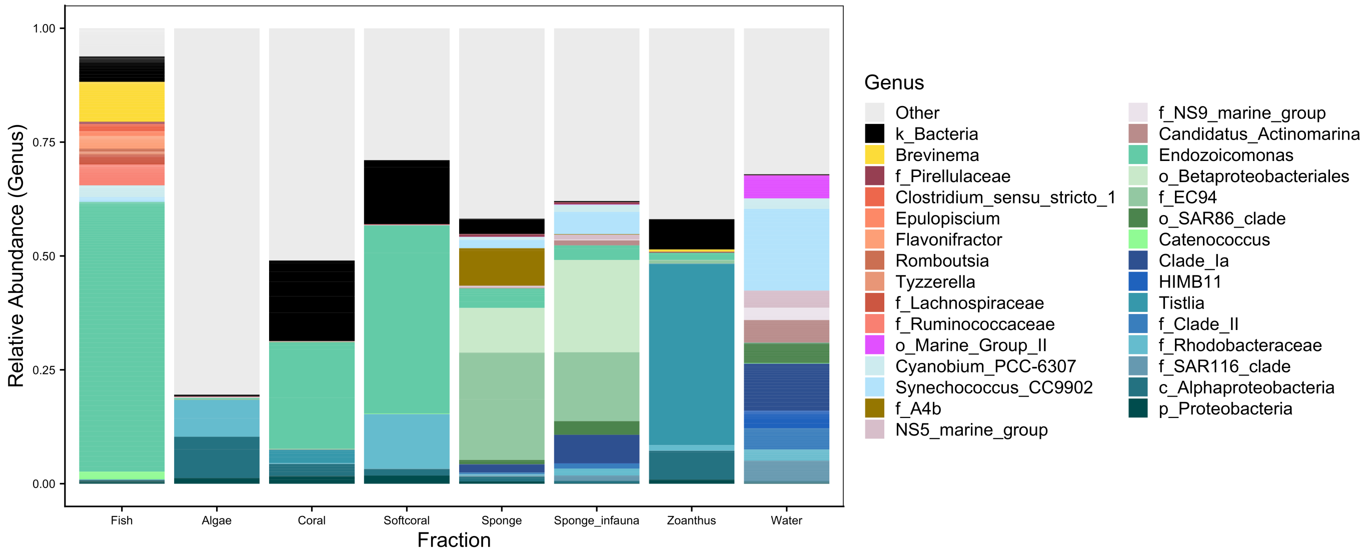

Figure S4. Mean relative read abundance of top microbial taxa across the different sample fractions comparing fish gut microbiome to potential prey items (i.e., algae, hard coral, soft coral, sponge, sponge infauna, zoanthid, anemone) and the surrounding seawater by phylum (a) and genus (b). The top taxa were selected for a (15 phyla) and b (30 genera), while pooling the remainder in the “other” category.

Shared microbial ASVs between fish guts and benthic taxa

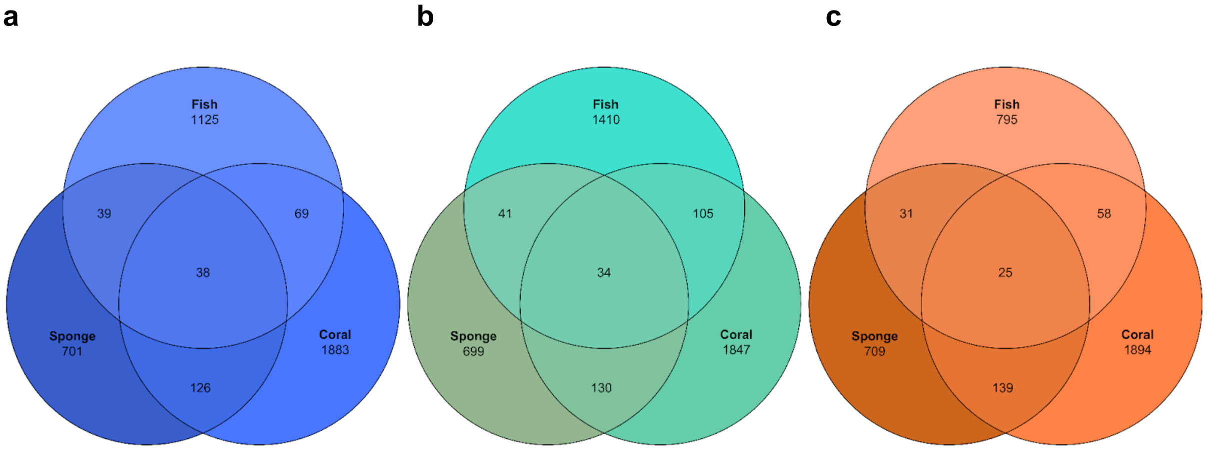

Figure S5. Shared ASVs among microbiomes of fish guts, coral (hard- and soft coral) and sponges based on the whole dataset. Fish gut samples are zone-specific, whereas both coral and sponge samples originated from various zones and were lumped due to the small sample size. (a) = outer bay, (b) = inner bay, (c) = inner bay disturbed.

Fish gut core microbiome

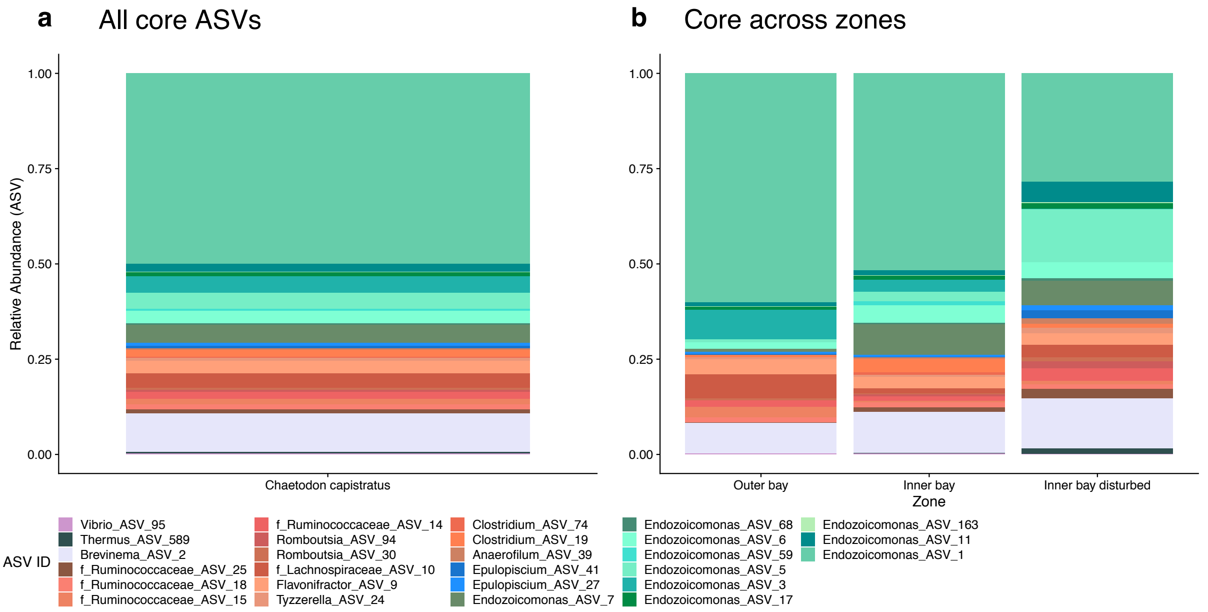

Figure S6. (a) Relative read abundance of the core bacterial community (*n* = 17 ASVs) in the gut of *Chaetodon capistratus* identified with Indicator Analysis by comparing 16S sequences found in fish guts to all other sample fractions (seawater and potential prey taxa) combined; (b) core microbiome community variation shown across three zones.

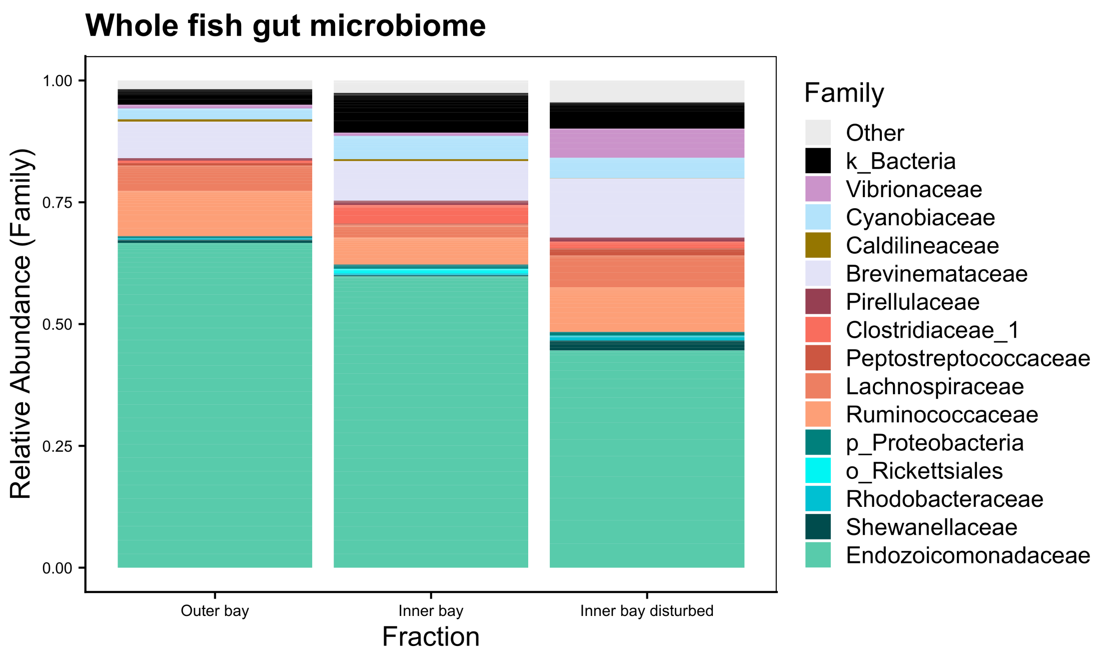

Figure S7. Mean relative read abundance of top microbial taxa in the whole fish gut microbiome across three reef zone by family. Remaining taxa were pooled in the “other” category.

a

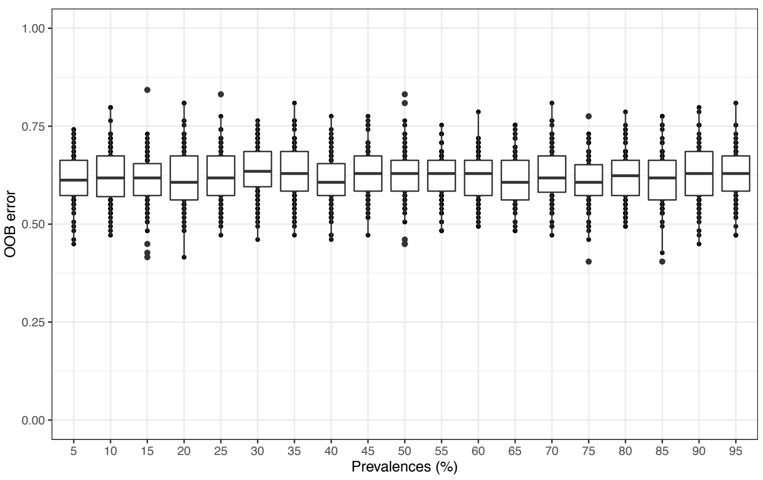

b

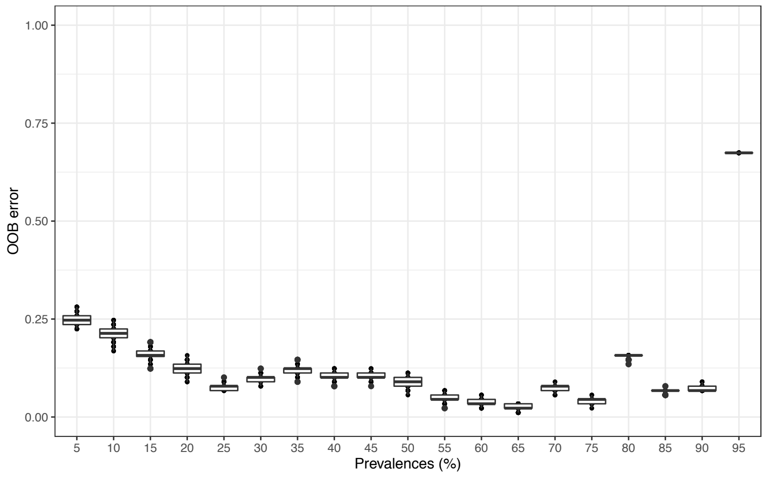

Figure S8. To validate PIME results, the algorithm assessed the likelihood of bias by simulating OBB error predictions. This was done by (i) by randomizing the group labels (i.e., zone identity) of the original dataset (17 ASVs) and using bootstrap aggregating (100 iterations) to perform a Monte Carlo simulation of Random Forest classifications on each filtering step (by 5 increments) generating boxplots for each prevalence interval at approximately the predicted best prevalence cut-off (65%) (a) and (ii) by repeating Random Forest OBB error estimations on the bootstrap aggregations of the filtered dataset at each prevalence interval (resulting in boxplots corresponding to the empirical data in Table S12) (b).

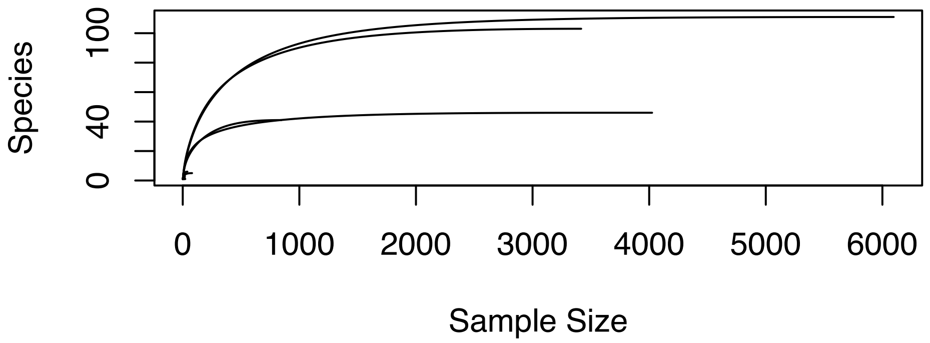

Figure S9. Rarefaction curves for 14 samples with fewer than 10,000 sequences. Curves plateau after a few thousand reads. To limit the variability in number of sequences between samples, these samples were removed from our dataset. We rarefied the remaining samples to even sequencing depth (*n* = 10,369 sequences).

II. Supplementary Tables

Table S1. Percent substrate cover across reef zones of the main substrate groups.

	Cover %		
**Reef Zone**	**Sponge**	**Dead hard coral**	**Live hard coral**
**Outer bay**	3.1	17.37	33.46
**Inner bay**	25.24	10.8	14.65
**Inner bay - disturbed**	23.53	40.1	0.35

Table S2. Core fish gut microbiome taxa identified with Indicator Analysis[3] comparing all fish gut microbial data combined to all other fractions combined (i.e. hard coral, soft coral, sponge, sponge infauna, zoanthid, anemone, algae, seawater). ASVs are ordered from the highest to lowest indicator value.

ASV ID	Indicator Value	*P*-Value	Frequency	Phylum	Class	Order	Family	Genus
ASV.1	0.996349277	0.001	111	Proteobacteria	Gammaproteobacteria	Oceanospirillales	Endozoicomonadaceae	Endozoicomonas
ASV.5	0.7413017	0.001	67	Proteobacteria	Gammaproteobacteria	Oceanospirillales	Endozoicomonadaceae	Endozoicomonas
ASV.6	0.730337079	0.001	65	Proteobacteria	Gammaproteobacteria	Oceanospirillales	Endozoicomonadaceae	Endozoicomonas
ASV.9	0.707730187	0.001	65	Firmicutes	Clostridia	Clostridiales	Ruminococcaceae	Flavonifractor
ASV.7	0.696629213	0.001	62	Proteobacteria	Gammaproteobacteria	Oceanospirillales	Endozoicomonadaceae	Endozoicomonas
ASV.14	0.696629213	0.001	62	Firmicutes	Clostridia	Clostridiales	Ruminococcaceae	NA
ASV.11	0.674157303	0.001	60	Proteobacteria	Gammaproteobacteria	Oceanospirillales	Endozoicomonadaceae	Endozoicomonas
ASV.2	0.659837502	0.001	71	Spirochaetes	Spirochaetia	Brevinematales	Brevinemataceae	Brevinema
ASV.18	0.572730393	0.001	52	Firmicutes	Clostridia	Clostridiales	Ruminococcaceae	NA
ASV.10	0.550260717	0.001	53	Firmicutes	Clostridia	Clostridiales	Lachnospiraceae	NA
ASV.17	0.539325843	0.001	48	Proteobacteria	Gammaproteobacteria	Oceanospirillales	Endozoicomonadaceae	Endozoicomonas
ASV.3	0.538432561	0.001	68	Proteobacteria	Gammaproteobacteria	Oceanospirillales	Endozoicomonadaceae	Endozoicomonas
ASV.27	0.505419537	0.002	46	Firmicutes	Clostridia	Clostridiales	Lachnospiraceae	Epulopiscium
ASV.68	0.447638938	0.001	41	Proteobacteria	Gammaproteobacteria	Oceanospirillales	Endozoicomonadaceae	Endozoicomonas
ASV.15	0.426966292	0.001	38	Firmicutes	Clostridia	Clostridiales	Ruminococcaceae	NA
ASV.30	0.370786517	0.003	33	Firmicutes	Clostridia	Clostridiales	Peptostreptococcaceae	Romboutsia
ASV.95	0.348314607	0.002	31	Proteobacteria	Gammaproteobacteria	Vibrionales	Vibrionaceae	Vibrio
ASV.94	0.34551753	0.004	32	Firmicutes	Clostridia	Clostridiales	Peptostreptococcaceae	Romboutsia
ASV.25	0.325842697	0.004	29	Firmicutes	Clostridia	Clostridiales	Ruminococcaceae	NA
ASV.19	0.314606742	0.003	28	Firmicutes	Clostridia	Clostridiales	Clostridiaceae_1	Clostridium_sensu_stricto_1
ASV.24	0.314606742	0.001	28	Firmicutes	Clostridia	Clostridiales	Lachnospiraceae	Tyzzerella
ASV.41	0.269662921	0.009	24	Firmicutes	Clostridia	Clostridiales	Lachnospiraceae	Epulopiscium
ASV.74	0.258426966	0.008	23	Firmicutes	Clostridia	Clostridiales	Clostridiaceae_1	Clostridium_sensu_stricto_2
ASV.163	0.251085004	0.005	24	Proteobacteria	Gammaproteobacteria	Oceanospirillales	Endozoicomonadaceae	Endozoicomonas
ASV.59	0.235955056	0.006	21	Proteobacteria	Gammaproteobacteria	Oceanospirillales	Endozoicomonadaceae	Endozoicomonas
ASV.589	0.235955056	0.006	21	Deinococcus-Thermus	Deinococci	Thermales	Thermaceae	Thermus
ASV.39	0.213483146	0.009	19	Firmicutes	Clostridia	Clostridiales	Ruminococcaceae	Anaerofilum

Table S3. Kruskal Wallis Rank Sum Test of Hill diversity[1,2]. Alpha diversity was measured using three metrics that put more or less weigh on common species (ASVs) (Hill numbers, {q = 0, 1, 2}) and Kruskal Wallis tests were used to test for significant differences in alpha diversity levels for each metric among reef zones and reefs respectively. Significance is shown in bold.

Microbiota	Factor	Diversity	Kruskal-Wallis χ^2	DF	*P*-Value
Whole	Zone	Observed q=0	3.494	2	0.174
		Shannon exponential q=1	10.996	2	**0.004**
		Simpsons multiplicative inverse q=2	8.634	2	**0.013**
Core	Zone	Observed	7.416	2	**0.025**
		Shannon exponential q=1	8.357	2	**0.015**
		Simpsons multiplicative inverse q=2	8.263	2	**0.016**
Whole	Reef	Observed q=0	9.916	8	0.271
		Shannon exponential q=1	13.543	8	0.094
		Simpsons multiplicative inverse q=2	10.529	8	0.23
Core	Reef	Observed	19.944	8	**0.011**
		Shannon exponential q=1	23.421	8	**0.003**
		Simpsons multiplicative inverse q=2	22.052	8	**0.005**

Table S4
Post-hoc Dunn test by zone with Benjamin Hochberg correction

Microbiota	Factor	Diversity	Zone	Z	*P*-Value	adjusted *P*-Value
Whole	Zone	Observed q=0	Inner bay-Inner bay disturbed	0.009	0.993	0.993
			Inner bay-Outer bay	1.701	0.089	0.267
			Inner bay disturbed-Outer bay	1.418	0.156	0.234
		Shannon exponential q=1	Inner bay-Inner bay disturbed	-1.416	0.157	0.157
			Inner bay-Outer bay	2.128	**0.033**	**0.05**
			Inner bay disturbed-Outer bay	3.201	**0.001**	**0.004**
		Simpsons multiplicative inverse q=2	Inner bay-Inner bay disturbed	-1.579	0.114	0.114
			Inner bay-Outer bay	1.587	0.113	0.169
			Inner bay disturbed-Outer bay	2.911	**0.004**	**0.011**
Core	Zone	Observed q=0	Inner bay-Inner bay disturbed	0.16	0.436	0.436
			Inner bay-Outer bay	2.535	**0.006**	**0.017**
			Inner bay disturbed-Outer bay	1.966	**0.025**	**0.049**
		Shannon exponential q=1	Inner bay-Inner bay disturbed	-1.633	0.102	0.154
			Inner bay-Outer bay	1.48	0.139	0.139
			Inner bay disturbed-Outer bay	2.875	**0.004**	**0.012**
		Simpsons multiplicative inverse q=2	Inner bay-Inner bay disturbed	-1.945	0.052	0.078
			Inner bay-Outer bay	1.106	0.269	0.269
			Inner bay disturbed-Outer bay	2.872	**0.004**	**0.012**

Table S5
Post-hoc Dunn test by reef with Benjamin Hochberg correction (only listing significantly different comparisons)

Microbiota	Factor	Diversity	Reef	Z	*P*-Value	adjusted *P*-Value
Core	Reef	Observed q=0	SCR-SIS	-3.525	**0.0002**	**0.008**
			SCR-ROL	3.441	**0.0003**	**0.01**
			SCR -PST	2.448	**0.007**	0.216
			SCR-RNW	3.493	**0.0002**	**0.008**
			SCR-PBL	1.758	**0.039**	1
			SCR-ALR	2.847	**0.002**	0.073
			SCR-CCR	2.837	**0.002**	0.073
			SCR-PPR	2.483	**0.007**	0.202
		Shannon exponential q=1	SCR-SIS	-2.83	**0.005**	**0.033**
			SCR-ROL	3.266	**0.001**	**0.01**
			SCR-ALR	2.252	**0.024**	0.11
			SCR-RNW	3.869	**0.0001**	**0.004**
			SCR-PBL	2.484	**0.013**	0.067
			PST-SCR	3.443	**0.001**	**0.01**
			SCR-CCR	2.794	**0.005**	**0.031**
			SCR-PPR	3.27	**0.001**	**0.013**
		Simpsons multiplicative inverse q=2	ALR-PST	-1.943	**0.052**	0.208
			ALR-RNW	-2.06	**0.039**	0.177
			SCR-SIS	-2.417	**0.016**	0.081
			SCR-ROL	3.046	**0.002**	**0.021**
			SCR-PBL	2.419	**0.016**	0.093
			SCR-PST	3.385	**0.001**	**0.013**
			SCR-RNW	3.742	**0.0002**	**0.007**
			SCR-CCR	2.667	**0.008**	0.055
			SCR-PPR	3.061	**0.002**	**0.027**

Table S6. Multivariate beta dispersion[4,5] of fish gut microbial communities compared among reef zones. Significance is shown in bold.

Metric	Model		Df	SumSq	MeanSq	F	N.Perm	Pr(>F)
Jaccard	Inner vs.	Groups	1	0.00124	0.0012389	0.34	10000	0.5637
	Outer bay	Residuals	87	0.31702	0.0036439			
	Low vs. high	Groups	1	0.000012	0.0000116	0.0027	10000	0.9614
	coral cover	Residuals	52	0.222844	0.0042855			
mod Gower	Inner vs.	Groups	1	0.051	0.050968	0.9796	10000	0.3213
	Outer bay	Residuals	87	4.5267	0.052031			
	Low vs. high	Groups	1	0.11257	0.112573	2.1817	10000	0.1467
	coral cover	Residuals	52	2.68311	0.051598			
Bray-Curtis	Inner vs.	Groups	1	0.27014	0.270138	13.215	10000	**5e-04 *****
	Outer bay	Residuals	87	1.77841	0.020441			
	Low vs. high	Groups	1	0.09116	0.091155	2.7183	10000	0.1066
	coral cover	Residuals	52	1.74376	0.033534			
Unifrac	Inner vs.	Groups	1	0.0058	0.0058003	0.9247	10000	0.3446
	Outer bay	Residuals	87	0.69462	0.0079841			
	Low vs. high	Groups	1	0.00025	0.0002453	0.0353	10000	0.8512
	coral cover	Residuals	52	0.36158	0.0069535			
Generalized Unifrac	Inner vs.	Groups	1	0.07265	0.072651	7.6237	10000	**0.006899 ****
	Outer bay	Residuals	87	0.82908	0.00953			
	Low vs. high	Groups	1	0.00005	0.0000468	0.005	10000	0.9426
	coral cover	Residuals	52	0.48636	0.0093531			
Weighted Unifrac	Inner vs.	Groups	1	0.02471	0.024714	4.8833	10000	**0.0268 ***
	Outer bay	Residuals	87	0.4403	0.0050609			
	Low vs. high	Groups	1	0.00176	0.0017639	0.2856	10000	0.6054
	coral cover	Residuals	52	0.32119	0.0061768			
Jaccard	3 habitat	Groups	2	0.00017	0.0000857	0.0208	10000	0.9775
	zones	Residuals	86	0.35484	0.0041261			
mod Gower	3 habitat	Groups	2	0.1586	0.07928	1.7449	10000	0.182
	zones	Residuals	86	3.9074	0.045435			
Bray-Curtis	3 habitat	Groups	2	0.27062	0.13531	5.9346	10000	**0.0033 ****
	zones	Residuals	86	1.96081	0.0228			
	Outside vs Inner disturbed							0.0007
Unifrac	3 habitat	Groups	2	0.0038	0.0018976	0.2856	10000	0.7483
	zones	Residuals	86	0.57141	0.0066443			
Generalized Unifrac	3 habitat	Groups	2	0.06929	0.034647	3.6283	10000	**0.0295 ***
	zones	Residuals	86	0.82122	0.009549			
	outside vs inner							0.0212
	outside vs inner disturbed							0.0381
Weighted Unifrac	3 Habitat	Groups	2	0.02588	0.0129393	2.561	10000	**0.07729 .**
	Zones	Residuals	86	0.43451	0.0050524			
	outside vs inner							0.0251

Signif. codes: 0 ‘***’ 0.001 ‘**’ 0.01 ‘*’ 0.05 ‘.’ 0.1 ‘ ’ 1

Table S7. Multivariate beta dispersion[4,5] of the core fish gut microbiome compared among reef zones. Significance is shown in bold.

Metric	Model		Df	SumSq	MeanSq	F	N.Perm	Pr(>F)
Jaccard	Inner vs.	Groups	1	0.00212	0.0021159	0.1517	10000	0.7032
	Outer bay	Residuals	87	1.21375	0.0139511			
	Low vs. high	Groups	1	0.00561	0.0056089	0.3921	10000	0.5372
	coral cover	Residuals	52	0.74391	0.014306			
mod Gower	Inner vs.	Groups	1	0.0179	0.017936	0.2367	10000	0.6254
	Outer bay	Residuals	87	6.5933	0.075785			
	Low vs. high	Groups	1	0.3177	0.3177	4.5296	10000	**0.0387 ***
	coral cover	Residuals	52	3.6472	0.07014			
Bray-Curtis	Inner vs.	Groups	1	0.23731	0.237309	10.907	10000	**0.0011 ****
	Outer bay	Residuals	87	1.89296	0.021758			
	Low vs. high	Groups	1	0.07669	0.076693	3.5233	10000	**0.06939 .**
	coral cover	Residuals	52	1.1319	0.021767			
Unifrac	Inner vs.	Groups	1	0.0547	0.054697	3.4941	10000	**0.06419 .**
	Outer bay	Residuals	87	1.3619	0.015654			
	Low vs. high	Groups	1	0.01119	0.011193	0.7669	10000	0.3815
	coral cover	Residuals	52	0.75896	0.014596			
Generalized Unifrac	Inner vs.	Groups	1	0.0082	0.0082037	0.3851	10000	0.5394
	Outer bay	Residuals	87	1.8534	0.0213034			
	Low vs. high	Groups	1	0.04683	0.046834	2.3263	10000	0.1372
	coral cover	Residuals	52	1.04689	0.020132			
Weighted Unifrac	Inner vs.	Groups	1	0.00466	0.0046557	0.1563	10000	0.7019
	Outer bay	Residuals	87	2.59206	0.0297938			
	Low vs. high	Groups	1	0.07048	0.070476	2.4078	10000	0.1297
	coral cover	Residuals	52	1.52204	0.02927			
Jaccard	3 habitat	Groups	2	0.00563	0.0028155	0.1903	10000	0.8311
	zones	Residuals	86	1.27258	0.0147974			
mod Gower	3 habitat	Groups	2	0.3199	0.15996	2.0397	10000	0.1333
	zones	Residuals	86	6.7443	0.078422			
	Inner disturbed vs inner							**0.036996**
Bray-Curtis	3 habitat	Groups	2	0.2554	0.127699	5.4236	10000	**0.005 ****
	zones	Residuals	86	2.0249	0.023545			
	Outside vs Inner disturbed							**0.0014**
	Inner disturbed vs inner							0.0675932
Unifrac	3 habitat	Groups	2	0.06817	0.034086	2.1679	10000	0.1203
	zones	Residuals	86	1.3522	0.015723			
Generalized Unifrac	3 habitat	Groups	2	0.05237	0.026185	1.2036	10000	0.2994
	zones	Residuals	86	1.87099	0.021756			
Weighted Unifrac	3 habitat	Groups	2	0.07351	0.036753	1.2255	10000	0.3012
	zones	Residuals	86	2.5791	0.029989			

Signif. codes: 0 '***' 0.001 '**' 0.01 '*' 0.05 '.' 0.1 ' ' 1

Table S8. Permutational Analysis of Variance (PERMANOVA)[6] results for the whole fish gut microbiome Differences among fish gut microbial communities were tested using three models: (1) among three zones (zone model); (2) between reefs located inside versus outside of the bay (position model) and (3) between reefs of differential coral cover levels inside of the bay (cover model). Post-hoc pairwise PERMANOVA[7] were calculated with Bonferroni corrected *P*-values for the whole (c) and core (d) communities. Significance is shown in bold.

PERMANOVA Whole fish gut microbiome

Distance	Model	Factor	Df	SumsOfSqs	MeanSqs	F.Model	R2	Pr(>F)
Jaccard	Zone/Reef	Zone	2	1.561	0.78028	2.0715	0.04506	**1.00E-04**
Jaccard		Zone:Reef	6	2.935	0.48922	1.2988	0.08476	1.00E-04
Jaccard	Position/Reef	Position	1	0.889	0.88946	2.361	0.02568	**2.00E-04**
Jaccard		Position:Reef	7	3.606	0.51521	1.3678	0.10414	1.00E-04
Jaccard	Cover/Reef	Cover	1	0.6711	0.6711	1.7386	0.03235	**0.0019**
Jaccard		Cover:Reef	4	2.0033	0.50082	1.3304	0.09657	0.0015
modGower	Zone/Reef	Zone	2	3.479	1.73941	3.0818	0.06451	**1.00E-04**
modGower		Zone:Reef	6	5.293	0.88213	1.5629	0.09815	2.00E-04
modGower	Position/Reef	Position	1	2.044	2.0439	3.6213	0.0379	**2.00E-04**
modGower		Position:Reef	7	6.728	0.9611	1.7028	0.12476	1.00E-04
modGower	Cover/Reef	Cover	1	1.435	1.43492	2.4181	0.04267	**0.0009999**
modGower		Cover:Reef	4	3.707	0.92679	1.5618	0.1102	0.0016998
Bray Curtis	Zone/Reef	Zone	2	2.2745	1.13725	4.4843	0.09217	**1.00E-04**
Bray Curtis		Zone:Reef	6	2.1136	0.35226	1.389	0.08565	0.049
Bray Curtis	Position/Reef	Position	1	1.2818	1.28176	5.0541	0.05194	**0.0003**
Bray Curtis		Position:Reef	7	3.1063	0.44376	1.7498	0.12588	0.0029
Bray Curtis	Cover/Reef	Cover	1	0.9927	0.99273	3.4134	0.0611	**0.0022**
Bray Curtis		Cover:Reef	4	1.2951	0.32377	1.1133	0.07971	0.2915
Unifrac	Zone/Reef	Zone	2	1.0915	0.54576	2.3968	0.05179	**0.0004**
Unifrac		Zone:Reef	6	1.7687	0.29479	1.2946	0.08392	0.0292
Unifrac	Position/Reef	Position	1	0.6944	0.69435	3.0494	0.03294	**0.0006999**
Unifrac		Position:Reef	7	2.1659	0.30941	1.3588	0.10276	0.0092991
Unifrac	Cover/Reef	Cover	1	0.3972	0.39716	1.69	0.03116	**0.0472**
Unifrac		Cover:Reef	4	1.0699	0.26748	1.1382	0.08393	0.1919
Generalized Unifrac	Zone/Reef	Zone	2	0.3157	0.157865	2.2534	0.04856	**0.008199**
Generalized Unifrac		Zone:Reef	6	0.5813	0.096875	1.3828	0.0894	0.055794
Generalized Unifrac	Position/Reef	Position	1	0.1855	0.185504	2.6479	0.02853	**0.0129**
Generalized Unifrac		Position:Reef	7	0.7115	0.10164	1.4508	0.10943	0.0283
Generalized Unifrac	Cover/Reef	Cover	1	0.1302	0.130226	1.5992	0.0294	0.1154
Generalized Unifrac		Cover:Reef	4	0.3903	0.097563	1.1981	0.08811	0.1997
Weighted Unifrac	Zone/Reef	Zone	2	0.06542	0.032708	1.9084	0.04175	0.07089
Weighted Unifrac		Zone:Reef	6	0.13032	0.02172	1.2673	0.08318	0.18758
Weighted Unifrac	Position/Reef	Position	1	0.03581	0.035813	2.0896	0.02286	0.09119
Weighted Unifrac		Position:Reef	7	0.15992	0.022846	1.333	0.10207	0.14389
Weighted Unifrac	Cover/Reef	Cover	1	0.0296	0.029603	1.3917	0.0259	0.229
Weighted Unifrac		Cover:Reef	4	0.09229	0.023073	1.0847	0.08075	0.3582

Table S9. Permutational Analysis of Variance (PERMANOVA)[6] results for the core fish gut microbiome Differences among fish gut microbial communities were tested using three models: (1) among three zones (zone model); (2) between reefs located inside versus outside of the bay (position model) and (3) between reefs of differential coral cover levels inside of the bay (cover model). Post-hoc pairwise PERMANOVA7 were calculated with Bonferroni corrected P-values for the whole (c) and core (d) communities. Significance is shown in bold.

PERMANOVA Core fish gut microbiome

Distance	Model	Factor	Df	SumsOfSqs	MeanSqs	F.Model	R2	Pr(>F)
Jaccard	Zone/Reef	Zone	2	1.7548	0.87738	5.1227	0.10103	**1.00E-04**
Jaccard		Zone:Reef	6	1.9124	0.31873	1.861	0.1101	0.0005999
Jaccard	Position/Reef	Position	1	1.2367	1.23675	7.2209	0.0712	**1.00E-04**
Jaccard		Position:Reef	7	2.4304	0.3472	2.0272	0.13993	2.00E-04
Jaccard	Cover/Reef	Cover	1	0.518	0.518	2.9928	0.05207	**0.0031**
Jaccard		Cover:Reef	4	1.1226	0.28065	1.6215	0.11284	0.0163
modGower	Zone/Reef	Zone	2	11.341	5.6703	4.4255	8.65E-02	**1.00E-04**
modGower		Zone:Reef	6	17.216	2.8694	2.2395	1.31E-01	1.00E-04
modGower	Position/Reef	Position	1	7.98	7.9798	6.228	0.06089	**1.00E-04**
modGower		Position:Reef	7	20.577	2.9396	2.2943	0.15701	1.00E-04
modGower	Cover/Reef	Cover	1	3.361	3.3608	2.5099	0.04429	**0.0115**
modGower		Cover:Reef	4	8.244	2.0609	1.5391	0.10865	0.0302
Bray Curtis	Zone/Reef	Zone	2	1.9772	0.98858	4.2749	0.08802	**1.00E-04**
Bray Curtis		Zone:Reef	6	1.9861	0.33102	1.4314	0.08841	0.05409
Bray Curtis	Position/Reef	Position	1	1.1449	1.14491	4.9509	0.05097	**0.0005999**
Bray Curtis		Position:Reef	7	2.8183	0.40262	1.741	0.12546	0.0053995
Bray Curtis	Cover/Reef	Cover	1	0.8323	0.83225	3.1407	0.05604	**0.005599**
Bray Curtis		Cover:Reef	4	1.2991	0.32478	1.2256	0.08748	0.184882
Unifrac	Zone/Reef	Zone	2	0.7624	0.3812	3.4988	0.07136	**0.0006999**
Unifrac		Zone:Reef	6	1.2049	0.20082	1.8431	0.11278	0.0079992
Unifrac	Position/Reef	Position	1	0.6341	0.63405	5.8195	0.05935	**1.00E-04**
Unifrac		Position:Reef	7	1.3332	0.19046	1.7481	0.12479	0.0103
Unifrac	Cover/Reef	Cover	1	0.1284	0.12835	1.2834	0.02381	0.2794
Unifrac		Cover:Reef	4	0.462	0.11549	1.1548	0.08569	0.3073
Generalized Unifrac	Zone/Reef	Zone	2	0.4435	0.22176	1.3724	0.0295	0.1942
Generalized Unifrac		Zone:Reef	6	1.6639	0.27732	1.7163	0.11068	0.0421
Generalized Unifrac	Position/Reef	Position	1	0.1747	0.1747	1.0812	0.01162	0.3097
Generalized Unifrac		Position:Reef	7	1.9327	0.27611	1.7087	0.12856	0.0346
Generalized Unifrac	Cover/Reef	Cover	1	0.2688	0.26882	1.5667	0.02899	0.1618
Generalized Unifrac		Cover:Reef	4	0.7682	0.19206	1.1193	0.08285	0.3236
Weighted Unifrac	Zone/Reef	Zone	2	0.2567	0.12835	1.0153	0.02215	0.38056
Weighted Unifrac		Zone:Reef	6	1.2182	0.20304	1.6061	0.10513	0.09289
Weighted Unifrac	Position/Reef	Position	1	0.0664	0.066447	0.52562	0.00573	0.60884
Weighted Unifrac		Position:Reef	7	1.4085	0.201211	1.59165	0.12154	0.08489
Weighted Unifrac	Cover/Reef	Cover	1	0.1903	0.19025	1.4289	0.0265	0.2238
Weighted Unifrac		Cover:Reef	4	0.5972	0.14929	1.1212	0.08319	0.3352

Table S10.
Pairwise PERMANOVA
Whole fish gut microbiome

Distance	Pairs	Df	SumsOfSqs	F.Model	R2	p.value	p.adjusted
Jaccard	Outer vs Inner bay	1	0.9179362	2.391052	0.03396813	**0.001**	**0.003**
Jaccard	Outer vs Inner dist.	1	0.7110085	1.852244	0.03439494	**0.003**	**0.009**
Jaccard	Inner vs Inner dist.	1	0.6710959	1.738583	0.03235261	**0.002**	**0.006**
modGower	Outer vs Inner bay	1	1.771492	3.2316	0.04536756	**0.001**	**0.003**
modGower	Outer vs Inner dist.	1	2.002315	3.31333	0.05990112	**0.001**	**0.003**
modGower	Inner vs Inner dist.	1	1.434917	2.3179	0.04267286	**0.002**	**0.006**
Bray Curtis	Outer vs Inner bay	1	0.7351019	3.061599	0.04308373	**0.012**	**0.036**
Bray Curtis	Outer vs Inner dist.	1	1.8030557	7.091213	0.12000453	**0.001**	**0.003**
Bray Curtis	Inner vs Inner dist.	1	0.9927345	3.38389	0.06109882	**0.004**	**0.012**
Unifrac	Outer vs Inner bay	1	0.7211911	3.130732	0.04401377	**0.001**	**0.003**
Unifrac	Outer vs Inner dist.	1	0.4669362	2.030862	0.03758708	**0.012**	**0.036**
Unifrac	Inner vs Inner dist.	1	0.397157	1.672233	0.03115639	0.056	0.168
GUnifrac	Outer vs Inner bay	1	0.1862079	2.693074	0.03809531	**0.021**	0.063
GUnifrac	Outer vs Inner dist.	1	0.1487629	2.294963	0.04226843	**0.019**	0.057
GUnifrac	Inner vs Inner dist.	1	0.1302265	1.575228	0.02940218	0.114	0.342
WUnifrac	Outer vs Inner bay	1	0.04234835	2.472368	0.0350828	0.063	0.189
WUnifrac	Outer vs Inner dist.	1	0.02331571	1.672923	0.03116885	0.136	0.408
WUnifrac	Inner vs Inner dist.	1	0.0296028	1.382646	0.02590066	0.227	0.681

Table S11.
Pairwise PERMANOVA
Core fish gut microbiome

Distance	Pairs	Df	SumsOfSqs	F.Model	R2	p.value	p.adjusted
Jaccard	Outer vs Inner bay	1	1.0424578	5.865352	0.079406	**0.001**	**0.003**
Jaccard	Outer vs Inner dist.	1	1.0227554	5.475954	0.09527383	**0.001**	**0.003**
Jaccard	Inner vs Inner dist.	1	0.5180037	2.856284	0.05206849	**0.007**	**0.021**
modGower	Outer vs Inner bay	1	6.22975	4.689234	0.06451071	**0.001**	**0.003**
modGower	Outer vs Inner dist.	1	7.254523	4.926	0.0865334	**0.001**	**0.003**
modGower	Inner vs Inner dist.	1	3.360785	2.409937	0.04429222	**0.019**	0.057
Bray Curtis	Outer vs Inner bay	1	0.6038367	2.763475	0.03905227	**0.012**	**0.036**
Bray Curtis	Outer vs Inner dist.	1	1.6436521	7.066327	0.11963376	**0.001**	**0.003**
Bray Curtis	Inner vs Inner dist.	1	0.8322521	3.087106	0.05604045	**0.005**	**0.015**
Unifrac	Outer vs Inner bay	1	0.4691397	3.860933	0.05372784	**0.004**	**0.012**
Unifrac	Outer vs Inner dist.	1	0.5200624	4.280836	0.07606205	**0.001**	**0.003**
Unifrac	Inner vs Inner dist.	1	0.1283554	1.268345	0.02381048	0.243	0.729
GUnifrac	Outer vs Inner bay	1	0.1198879	0.7424537	0.01080051	0.497	1
GUnifrac	Outer vs Inner dist.	1	0.306756	1.734408	0.03227742	0.142	0.426
GUnifrac	Inner vs Inner dist.	1	0.2688158	1.5524443	0.02898923	0.164	0.492
WUnifrac	Outer vs Inner bay	1	0.05790827	0.4694963	0.00685702	0.664	1
WUnifrac	Outer vs Inner dist.	1	0.15775916	1.1256392	0.02118825	0.328	0.984
WUnifrac	Inner vs Inner dist.	1	0.1902502	1.4156865	0.0265032	0.213	0.639

Table S12. Prevalence Interval for Microbiome Evaluation (PIME)[8].

Using RandomForest, the algorithm determined the prevalence level, which provided the best model to predict differences among the three reef zone microbial communities. The table lists the output of the best prevalence function (PIME R package)[8] with the out of bag (OBB) error rate for each prevalence interval and the AVSs retained in the dataset as well as the associated number of remaining sequences (Nseq). The prevalence bin chosen for the present study (65%) is depicted in bold.

Prevalence Interval	OBB error rate %	ASVs	Nseqs
5%	22.47	1143	897208
10%	20.22	266	844546
15%	13.48	118	799278
20%	10.11	76	784277
25%	8.99	57	770883
30%	8.99	42	753340
35%	11.24	36	744032
40%	10.11	36	739985
45%	8.99	30	732452
50%	6.74	23	706945
55%	4.49	21	674387
60%	3.37	18	600083
**65%**	**2.25**	**17**	**561593**
70%	7.87	10	496649
75%	4.49	7	457736
80%	15.73	4	424993
85%	6.74	4	418457
90%	6.74	3	405206
95%	67.42	1	365907

Table S13. Prevalence Interval for Microbiome Evaluation (PIME)[8].
Results for the PIME filtered community comprising 17 ASVs that are responsible for discriminating the three reef zone communities. Based on RandomForest, the algorithm calculated (i) Mean Decrease Accuracy where higher values indicate the importance of taxa in causing differences among the three reef zones, while positive values indicate ASVs truly contributed to discern zones – the importance values are reported across all decision trees and broken up by respective reef zones and (ii) Mean Decrease Gini (or mean decrease impurity), a measure of how reliable a variable predicts the model and refers to node splitting in a decision tree, where the algorithm looks for the feature (here ASV) to split where the split results in the lowest node impurity for the most optimal model.

ASV	Inner bay	Inner bay disturbed	Outer bay	Mean Decrease Accuracy	Mean Decrease Gini
ASV6	0.056103703	0.021597619	0.174855597	0.093632368	6.740867519
ASV7	0.08671229	0.088669841	0.099411424	0.09076883	7.98316477
ASV3	0.005125934	0.160498485	0.050850058	0.053926743	5.720833825
ASV9	0.001270734	0.011368326	0.096647221	0.040011572	3.573840343
ASV42	0.02385976	0.043803535	0.049101124	0.037602644	4.043649685
ASV27	0.024402986	0.03225267	0.055083597	0.036728284	3.320584212
ASV41	0.021518957	0.062058802	0.024365059	0.031886283	3.95981884
ASV2	0.023511911	0.029188745	0.038238022	0.030100204	3.120207217
ASV10	0.008987344	0.066901371	0.027017528	0.028201424	3.030194539
ASV4	0.018321354	0.030409235	0.037108942	0.02782255	2.688240385
ASV14	0.054866006	0.012504834	0.007273479	0.026189242	2.776662362
ASV38	0.019521715	0.029386797	0.018296985	0.020691546	2.628507801
ASV94	0.010840017	0.032298196	0.018855767	0.018416502	2.371929934
ASV1	0.00192246	0.018927056	0.011505233	0.008862312	1.360170402
ASV11	0.000392347	0.051716667	-0.003903	0.008319567	1.061015645
ASV8	0.00148597	9.00E-05	0.012271321	0.005533648	1.131796017
ASV5	3.85E-05	0.005655916	0.005317374	0.003572141	0.954048098

Table S14. Prevalence Interval for Microbiome Evaluation (PIME)[8]. Taxonomy table of the PIME filtered community comprising 17 ASVs that are responsible for discriminating the three reef zone communities.

ASV	Kingdom	Phylum	Class	Order	Family	Genus
ASV1	Bacteria	Proteobacteria	Gammaproteobacteria	Oceanospirillales	Endozoicomonadaceae	Endozoicomonas
ASV2	Bacteria	Spirochaetes	Spirochaetia	Brevinematales	Brevinemataceae	Brevinema
ASV3	Bacteria	Proteobacteria	Gammaproteobacteria	Oceanospirillales	Endozoicomonadaceae	Endozoicomonas
ASV4	Bacteria	Cyanobacteria	Oxyphotobacteria	Synechococcales	Cyanobiaceae	Synechococcus_CC9902
ASV5	Bacteria	Proteobacteria	Gammaproteobacteria	Oceanospirillales	Endozoicomonadaceae	Endozoicomonas
ASV6	Bacteria	Proteobacteria	Gammaproteobacteria	Oceanospirillales	Endozoicomonadaceae	Endozoicomonas
ASV7	Bacteria	Proteobacteria	Gammaproteobacteria	Oceanospirillales	Endozoicomonadaceae	Endozoicomonas
ASV8	Bacteria	Cyanobacteria	Oxyphotobacteria	Synechococcales	Cyanobiaceae	Cyanobium_PCC-6307
ASV9	Bacteria	Firmicutes	Clostridia	Clostridiales	Ruminococcaceae	Flavonifractor
ASV11	Bacteria	Proteobacteria	Gammaproteobacteria	Oceanospirillales	Endozoicomonadaceae	Endozoicomonas
ASV27	Bacteria	Firmicutes	Clostridia	Clostridiales	Lachnospiraceae	Epulopiscium
ASV42	Bacteria	Planctomycetes	Planctomycetacia	Pirellulales	Pirellulaceae	NA
ASV10	Bacteria	Firmicutes	Clostridia	Clostridiales	Lachnospiraceae	NA
ASV14	Bacteria	Firmicutes	Clostridia	Clostridiales	Ruminococcaceae	NA
ASV41	Bacteria	Firmicutes	Clostridia	Clostridiales	Lachnospiraceae	Epulopiscium
ASV94	Bacteria	Firmicutes	Clostridia	Clostridiales	Peptostreptococcaceae	Romboutsia
ASV38	Bacteria	Proteobacteria	Alphaproteobacteria	Rhodobacterales	Rhodobacteraceae	Ruegeria

Table S15. Number of fish individuals sampled per reef and reef site geographic coordinates. The reef zone category shows the assignment of individual reefs to zones.

Reef	Reef name	Latitude	Longitude	Reef Zone	Number of fishes sampled
ALR	Almirante	N 09.28998	W 82.34308	Inner bay	12
ROL	Cayo Roldan	N 09.21478	W 82.32454	Inner bay	15
SIS	Cayo Hermanas	N 09.26751	W 82.35178	Inner bay	8
PBL	Punta Puebla	N 09.36665	W 82.29124	Inner bay disturbed	6
PST	Punta STRI	N 09.34885	W 82.26292	Inner bay disturbed	5
RNW	Runway	N 09.34193	W 82.25997	Inner bay disturbed	8
CCR	Cayo Corales	N 09.26747	W 82.11983	Outer bay	16
PPR	Popa	N 09.23344	W 82.11189	Outer bay	7
SCR	Salt Creek	N 09.28002	W 82.10175	Outer bay	12

Table S16. Index PCR Primers

Primer name	Illumina flow cell adapter sequence	Index sequence	Locus specific primer
SC501F	AATGATACGGCGACCACCGAGATCTACAC	ACGACGTG	ACACTCTTTCCCTACACGAC
SC502F	AATGATACGGCGACCACCGAGATCTACAC	ATATACAC	ACACTCTTTCCCTACACGAC
SC503F	AATGATACGGCGACCACCGAGATCTACAC	CGTCGCTA	ACACTCTTTCCCTACACGAC
SC504F	AATGATACGGCGACCACCGAGATCTACAC	CTAGAGCT	ACACTCTTTCCCTACACGAC
SC505F	AATGATACGGCGACCACCGAGATCTACAC	GCTCTAGT	ACACTCTTTCCCTACACGAC
SC506F	AATGATACGGCGACCACCGAGATCTACAC	GACACTGA	ACACTCTTTCCCTACACGAC
SC507F	AATGATACGGCGACCACCGAGATCTACAC	TGCGTACG	ACACTCTTTCCCTACACGAC
SC508F	AATGATACGGCGACCACCGAGATCTACAC	TAGTGTAG	ACACTCTTTCCCTACACGAC
SD501F	AATGATACGGCGACCACCGAGATCTACAC	AAGCAGCA	ACACTCTTTCCCTACACGAC
SD502F	AATGATACGGCGACCACCGAGATCTACAC	ACGCGTGA	ACACTCTTTCCCTACACGAC
SD503F	AATGATACGGCGACCACCGAGATCTACAC	CGATCTAC	ACACTCTTTCCCTACACGAC
SD504F	AATGATACGGCGACCACCGAGATCTACAC	TGCGTCAC	ACACTCTTTCCCTACACGAC
SD505F	AATGATACGGCGACCACCGAGATCTACAC	GTCTAGTG	ACACTCTTTCCCTACACGAC
SD506F	AATGATACGGCGACCACCGAGATCTACAC	CTAGTATG	ACACTCTTTCCCTACACGAC
SD507F	AATGATACGGCGACCACCGAGATCTACAC	GATAGCGT	ACACTCTTTCCCTACACGAC
SD508F	AATGATACGGCGACCACCGAGATCTACAC	TCTACACT	ACACTCTTTCCCTACACGAC
SC701R	CAAGCAGAAGACGGCATACGAGAT	ACCTACTG	GTGACTGGAGTTCAGACGTGTGCTCTTCCGATCT
SC702R	CAAGCAGAAGACGGCATACGAGAT	AGCGCTAT	GTGACTGGAGTTCAGACGTGTGCTCTTCCGATCT
SC703R	CAAGCAGAAGACGGCATACGAGAT	AGTCTAGA	GTGACTGGAGTTCAGACGTGTGCTCTTCCGATCT
SC704R	CAAGCAGAAGACGGCATACGAGAT	CATGAGGA	GTGACTGGAGTTCAGACGTGTGCTCTTCCGATCT
SC705R	CAAGCAGAAGACGGCATACGAGAT	CTAGCTCG	GTGACTGGAGTTCAGACGTGTGCTCTTCCGATCT
SC706R	CAAGCAGAAGACGGCATACGAGAT	CTCTAGAG	GTGACTGGAGTTCAGACGTGTGCTCTTCCGATCT
SC707R	CAAGCAGAAGACGGCATACGAGAT	GAGCTCAT	GTGACTGGAGTTCAGACGTGTGCTCTTCCGATCT
SC708R	CAAGCAGAAGACGGCATACGAGAT	GGTATGCT	GTGACTGGAGTTCAGACGTGTGCTCTTCCGATCT
SC709R	CAAGCAGAAGACGGCATACGAGAT	GTATGACG	GTGACTGGAGTTCAGACGTGTGCTCTTCCGATCT
SC710R	CAAGCAGAAGACGGCATACGAGAT	TAGACTGA	GTGACTGGAGTTCAGACGTGTGCTCTTCCGATCT
SC711R	CAAGCAGAAGACGGCATACGAGAT	TCACGATG	GTGACTGGAGTTCAGACGTGTGCTCTTCCGATCT
SC712R	CAAGCAGAAGACGGCATACGAGAT	TCGAGCTC	GTGACTGGAGTTCAGACGTGTGCTCTTCCGATCT
SD701R	CAAGCAGAAGACGGCATACGAGAT	ACCTAGTATGCTCTTC	GTGACTGGAGTTCAGACGTG
SD702R	CAAGCAGAAGACGGCATACGAGAT	ACGTACGTTGCTCTTC	GTGACTGGAGTTCAGACGTG
SD703R	CAAGCAGAAGACGGCATACGAGAT	ATATCGCGTGCTCTTC	GTGACTGGAGTTCAGACGTG
SD704R	CAAGCAGAAGACGGCATACGAGAT	CACGATAGTGCTCTTC	GTGACTGGAGTTCAGACGTG
SD705R	CAAGCAGAAGACGGCATACGAGAT	CGTATCGCTGCTCTTC	GTGACTGGAGTTCAGACGTG
SD706R	CAAGCAGAAGACGGCATACGAGAT	CTGCGACTTGCTCTTC	GTGACTGGAGTTCAGACGTG
SD707R	CAAGCAGAAGACGGCATACGAGAT	GCTGTAAC	GTGACTGGAGTTCAGACGTG
SD708R	CAAGCAGAAGACGGCATACGAGAT	GGACGTTA	GTGACTGGAGTTCAGACGTG
SD709R	CAAGCAGAAGACGGCATACGAGAT	GGTCGTAG	GTGACTGGAGTTCAGACGTG
SD710R	CAAGCAGAAGACGGCATACGAGAT	TAAGTCTC	GTGACTGGAGTTCAGACGTG
SD711R	CAAGCAGAAGACGGCATACGAGAT	TACACAGT	GTGACTGGAGTTCAGACGTG
SD712R	CAAGCAGAAGACGGCATACGAGAT	TTGACGCA	GTGACTGGAGTTCAGACGTG

III. Supplementary Methods

DNA extraction of gut content

The gastrointestinal tract of each fish was opened longitudinally to isolate the digesta and the mucosa by lightly scraping the intestinal epithelium. Between 0.05 and 0.25 g of tissue was used for DNA extraction using the Qiagen Powersoil DNA isolation kit following the manufacturers instructions with minor modifications. To improve tissue lysis, 20 µL of Proteinase K (0.4 $mg.mL^{-1}$) was added into eppendorf tubes containing power beads and solution. Samples were briefly vortexed and incubated in a shaking incubator (1000 rpm) at 60°C for 15 minutes (min). Samples were vortexed for 5 min using a Vortex Genie 2 (Scientific Industries) with vortex adapter and incubated at 60°C for 1 hour and 45 min (1000 rpm) for a total of 2 hours incubation time. DNA was eluted in 100 ul buffer (Qiagen PowerSoil kit C6 solution) and DNA concentration was quantified with a Qubit Fluorometer (dsDNA High-Sensitivity Assay Kit, Invitrogen, Life Technologies). DNA extractions of the invertebrate and macroalgal tissues (0.25g per sample) were conducted using the same modified protocol.

PCR protocols

The first PCR amplification was performed in a total volume of 12.5 µL with 0.2 µL of 10 millimolars (mM) forward primer 515F (5' GTGYCAGCMGCCGCGGTAA 3'; [9], 0.2 µL of 10 mM reverse primer 806R (5' GGACTACNVGGGTWTCTAAT 3'; [10]), 5 µL of "5 PRIME Hot Master Mix (2.5x)" solution, 5.1 µL of nuclease-free water and 2µL of genomic DNA extract. This combination of primers has been recommended for marine microbial studies [11]. Primers were phased with heterogeneity spacers to increase the per base variability during Illumina sequencing [12]. PCR cycling conditions were 94°C for 3 min, followed by 35 cycles at 94°C for 45 s, 50°C for 1 min and 72°C for 1 min 30 sec and a final elongation step at 72°C for 10 min. Each sample was amplified three times independently and the product checked on 1.5% agarose gel. Triplicate PCRs were then pooled and purified using paramagnetic beads (KAPA Pure Beads) at a ratio sample:beads of 1:1.6. Purified PCR products were quantified using a Qubit Fluorometer and diluted to 5 nanograms per microlitre ($ng.\mu L^{-1}$). These dilutions were used in a second PCR to add unique combinations of dual index Illumina sequencing adaptors to each sample. Each PCR amplification was performed in a total volume of 11.5 µL with 1µL of 2.5 mM Forward indexed Illumina primer, 1 µL of 2.5 mM Reverse indexed Illumina primer, 5 µL of "5 PRIMER Hot Master Mix (2.5x)" solution, 3.5 µL of nuclease-free water and 1µL of diluted DNA. Here, cycling conditions were: 94°C, 3 min, followed by 6 cycles of 94°C for 45 s, 50°C for 1 min and 72°C for 1 min 30 s; and a final elongation step at 72°C for 10 min. Unique combinations of 16 unique Forward and 24 unique Reverse Illumina indexed primers were used in order to allow multiplexing of all samples (Table S16). Finally, an equal volume of indexed PCR product for each sample was mixed into a single tube. The pool was purified two successive times with paramagnetic beads at a ratio bead:sample of 1:1 to remove leftover primers and primer dimers.

Supplementary References

1. Alberdi, A. & Gilbert, M. T. P. A guide to the application of Hill numbers to DNA-based diversity analyses. *Mol. Ecol. Resour.* **19**, 1755–0998.13014 (2019).
2. Hill, M. O. Diversity and Evenness: A unifying notation and its consequences. *Ecology* **54**, 427–432 (1973).
3. Dufrêne, M. & Legendre, P. Species assemblages and indicator species: the need for a flexible asymmetrical approach. *Ecol. Monogr.* **67**, 345–366 (1997).
4. Anderson, M. J. Distance-based tests for homogeneity of multivariate dispersions. *Biometrics* **62**, 245–253 (2006).
5. Anderson, M. J., Ellingsen, K. E. & McArdle, B. H. Multivariate dispersion as a measure of beta diversity. *Ecol. Lett.* **9**, 683–693 (2006).
6. Anderson, M. J. A new method for non-parametric multivariate analysis of variance. *Austral Ecol.* **26**, 32–46 (2001).
7. Martinez Arbizu, P. pairwiseAdonis: Pairwise multilevel comparison using adonis. R package version 0.3. (2019). Available at: https://github.com/pmartinezarbizu/pairwiseAdonis.
8. Roesch, L. F. W. *et al.* PIME: A package for discovery of novel differences among microbial communities. *Mol. Ecol. Resour.* **20**, 415–428 (2020).
9. Parada, A. E., Needham, D. M. & Fuhrman, J. A. Every base matters: assessing small subunit rRNA primers for marine microbiomes with mock communities, time series and global field samples. *Environ. Microbiol.* **18**, 1403–1414 (2016).
10. Apprill, A., McNally, S., Parsons, R. & Weber, L. Minor revision to V4 region SSU rRNA 806R gene primer greatly increases detection of SAR11 bacterioplankton. *Aquat. Microb. Ecol.* **75**, 129–137 (2015).
11. Walters, W. *et al.* Improved Bacterial 16S rRNA Gene (V4 and V4-5) and fungal Internal Transcribed Spacer marker gene primers for microbial community surveys. *mSystems* **1**, (2016).
12. Fadrosh, D. W. *et al.* An improved dual-indexing approach for multiplexed 16S rRNA gene sequencing on the Illumina MiSeq platform. *Microbiome* **2**, 6 (2014).
