# Supplementary material for: The gut microbiome variability of a butterflyfish increases on severely degraded Caribbean reefs
Source: Commun Biol. 2022 Jul 30;5:770. doi: 10.1038/s42003-022-03679-0 (PMC9338936; doi:10.1038/s42003-022-03679-0)
Supplement: Supplementary file 2 — Reporting Summary [file 42003_2022_3679_MOESM2_ESM.pdf]

nature research

Corresponding author(s): Friederike Clever

Last updated by author(s): 2022 05 20

Reporting Summary

Nature Research wishes to improve the reproducibility of the work that we publish. This form provides structure for consistency and transparency in reporting. For further information on Nature Research policies, see our Editorial Policies and the Editorial Policy Checklist.

Statistics

For all statistical analyses, confirm that the following items are present in the figure legend, table legend, main text, or Methods section.

n/a	Confirmed	
☐	☒	The exact sample size (*n*) for each experimental group/condition, given as a discrete number and unit of measurement
☐	☒	A statement on whether measurements were taken from distinct samples or whether the same sample was measured repeatedly
☐	☒	The statistical test(s) used AND whether they are one- or two-sided<br>*Only common tests should be described solely by name; describe more complex techniques in the Methods section.*
☐	☒	A description of all covariates tested
☐	☒	A description of any assumptions or corrections, such as tests of normality and adjustment for multiple comparisons
☐	☒	A full description of the statistical parameters including central tendency (e.g. means) or other basic estimates (e.g. regression coefficient) AND variation (e.g. standard deviation) or associated estimates of uncertainty (e.g. confidence intervals)
☐	☒	For null hypothesis testing, the test statistic (e.g. *F*, *t*, *r*) with confidence intervals, effect sizes, degrees of freedom and *P* value noted<br>*Give P values as exact values whenever suitable.*
☒	☐	For Bayesian analysis, information on the choice of priors and Markov chain Monte Carlo settings
☐	☒	For hierarchical and complex designs, identification of the appropriate level for tests and full reporting of outcomes
☒	☐	Estimates of effect sizes (e.g. Cohen's *d*, Pearson's *r*), indicating how they were calculated

Our web collection on statistics for biologists contains articles on many of the points above.

Software and code

Policy information about availability of computer code

Data collection	Photographic quadrats of the coral reef benthos were analyzed on the CoralNet platform. Beijbom et al. Towards Automated Annotation of Benthic Survey Images: Variability of Human Experts and Operational Modes of Automation. PLoSOne 10, e0130312 (2015).
Data analysis	Code: https://bocasbiome.github.io/ R version 3.6.1; Phyloseq version 1.28.0; DADA2 version 1.12.1 all other R packages are reported in the manuscript.

For manuscripts utilizing custom algorithms or software that are central to the research but not yet described in published literature, software must be made available to editors and reviewers. We strongly encourage code deposition in a community repository (e.g. GitHub). See the Nature Research guidelines for submitting code & software for further information.

Data

Policy information about availability of data

All manuscripts must include a data availability statement. This statement should provide the following information, where applicable:

- Accession codes, unique identifiers, or web links for publicly available datasets
- A list of figures that have associated raw data
- A description of any restrictions on data availability

Sequencing data has been submitted to the NCBI Short Read Archive (SRA) database (https://www.ncbi.nlm.nih.gov/sra) under project number PRJNA718434 ID: 718434. Raw data are available on Dryad Digital Repository https://doi.org/10.5061/dryad.m905qfv28. Source code is available at https://github.com/bocasbiome/web/

Field-specific reporting

Please select the one below that is the best fit for your research. If you are not sure, read the appropriate sections before making your selection.

☐ Life sciences ☐ Behavioural & social sciences ☒ Ecological, evolutionary & environmental sciences

For a reference copy of the document with all sections, see nature.com/documents/nr-reporting-summary-flat.pdf

Ecological, evolutionary & environmental sciences study design

All studies must disclose on these points even when the disclosure is negative.

Study description	We analyzed changes in fish gut microbiomes of the foureye butterflyfish (Chaetodon capistratus) (n = 89) using Illumina high-throughput sequencing of the 16S ribosomal rRNA gene. We compared gut microbial communities among fish occuring on coral reefs featuring various levels of coral cover across a disturbed bay at Bocas del Toro, Panama. The microbial analysis was conducted on the levels of Amplicon Sequence Variants (ASVs). A subset of the whole microbiome (i.e., 'core microbiome') was identified for the overall fish population (n = 89) by comparing fish microbiomes and microbial communities of their surrounding environment combined i.e., seawater (four liters at each of nine reefs) and potential prey such as algae, anthozoans and sponges (n = 23), using Indicator Analysis. See schematic diagram of our workflow at https://bocasbiome.github.io/ (or see Fig. 7) for more details.<br>For the spatial comparison a total of nine reefs were grouped into three habitat zones: 'outer bay', 'inner bay', 'inner bay disturbed'. Benthic community cover was estimated from 100 x 70 cm photographic quadrats along three transects per reef (total number of transects: 27) with 10 quadrats per transect (total number of quadrats: 270). Fish focal species density, and overall fish and invertebrate community composition were assessed using visual censuses along the same transects.<br>We tested for significant differences among fish gut microbial communities in terms of i) alpha-diversity (hill diversity) among reefs and reef zones (Kruskal Wallis test with post-hoc Dunn test); ii) beta-diversity using a nested approach among (a) the two zones inside of the bay (n = 6 reefs) and the outer bay zone (n = 3 reefs), (b) the three zones (n = 9 reefs), (c) between the two zones located inside of the bay (n = 3 inner bay reefs and n = 3 inner bay disturbed reefs) using PERMANOVA with post-hoc pairwise PERMANOVA. Differences in multivariate beta-dispersion were tested using PERMDISP2 with the same nested approach as described for beta-diversity.<br>These comparative analyses were conducted for both the whole fish gut microbiome and the core fish gut microbiome. Additionally, we compared whole fish gut microbiomes among habitat zones by filtering for prevalent ASVs characterizing each zone using a random forest approach.
Research sample	89 fish gut microbiome samples of Chaetodon capistratus from the Bahia Amirante, Bocas del Toro Panama. Plus nine environmental DNA (eDNA) seawataer samples (one sample from each of nine reefs) and 23 samples of benthic biota (algae, corals, sponges) obtained from inner bay and outer bay reefs, both for microbiome analysis.
Sampling strategy	We employed a Resource Equation approach aiming for 10 - 20 residual degrees of freedom for each of our tests. So that if making multiple two group comparisons, a minimum of 10 samples per group provides residual degrees of freedom of 18 and thus plenty of room for blocking and covariates.
Data collection	Following a protocol of fish capture and euthanization approved by the Smithsonian Tropical Research Institute's Institutional Animal Care and Use Committee (IACUC), fish were collected by spearfishing in February and March 2018. Within the same time frame, small pieces of benthic species (potential fish prey items) were collected by hand using gloves. Seawater samples (each 4 L) were collected above the reef substratum and filtered through a 0.22 µm nitrocellulose membrane (Millipore). Fish and benthic species were collected by Friederike Clever, Lucia Rodriguez, Jarrod Scott, Matthieu Leray. Water was collected by Matthieu Leray. Ross Whippo conducted the fish survey and Clare Fieseler took photos of the benthos. Catalina Guerra Rodriguez and Joan Antaneda analyzed the photo quadrats on the CoralNet platform.
Timing and spatial scale	Data was colected across the Bahia Almirante at Bocas del Toro Panama, a semi-enclosed bay of approx. 450 km2. Collection sites comprised reefs located at the mouth of the bay and reef located at two different locations within the bay. Study reefs were separated from each other by deeper, non-reef habitats. Fish and environmental samples were collected between February-March 2018. Visual surveys of benthic cover and focal fish species densities were conducted between May and June 2016.
Data exclusions	Illumina Miseq generated sequences were quality filtered using DADA2 omitting sequences with more than two expected errors (maxEE = 2), or at least one ambiguous nucleotide (maxN = 0), or at least one base with a high probability of erroneous assignment (truncQ = 2). Fourteen samples containing few sequences (<10,000) were removed from the dataset.
Reproducibility	We make the raw data available and created a website (https://bocasbiome.github.io/) where we provide all code in an easy to follow order and with explanations. Furthermore, we provide our laboratory protocols and exact versions of R packages, databases and software in the article.
Randomization	We grouped individual reefs (n = 9) into habitat zones (n = 3) depending on geographic location and level of coral cover.
Blinding	Fish gut samples were randomly drawn from a pool of samples from all sites for DNA extraction and each sample was a assigned a DNA-Identifier number independent of site after extraction.

Did the study involve field work? ☒ Yes ☐ No

Field work, collection and transport

Field conditions	Fish collection was conducted during wet season.
Location	Bahia Almirante at Bocas del Toro, Panama located at the Caribbean Sea; Lat 9.271457° Long -82.218573°. Sampling was conducted at shallow reefs between 1 - 4 m depth.
Access & import/export	Our fish sampling protocol was approved by the Institutional Animal Care and Use Committee of the Smithsonian Tropical Research Institute (IACUC). A research permit was issued by the Ministerio de Ambiente Panamá (No. SE/A-113-17).
Disturbance	We chose a common reef fish as our study species with stable populations across the Caribbean including our study area, and which is listed as 'least concern' by the IUCN Red List. Our fish capture and euthanization protocol was optimised to reduce stress and pain on the collected animals and approved by the Institutional Animal Care and Use Committee of the Smithsonian Tropical Research Institute (IACUC). Upon capture, fish were immediately brought to the boat, anesthetized with clove oil and placed on ice.

Reporting for specific materials, systems and methods

We require information from authors about some types of materials, experimental systems and methods used in many studies. Here, indicate whether each material, system or method listed is relevant to your study. If you are not sure if a list item applies to your research, read the appropriate section before selecting a response.

Materials & experimental systems

n/a	Involved in the study	
☒	☐	Antibodies
☒	☐	Eukaryotic cell lines
☒	☐	Palaeontology and archaeology
☐	☒	Animals and other organisms
☒	☐	Human research participants
☒	☐	Clinical data
☒	☐	Dual use research of concern

Methods

n/a	Involved in the study	
☒	☐	ChIP-seq
☒	☐	Flow cytometry
☒	☐	MRI-based neuroimaging

Animals and other organisms

Policy information about studies involving animals; ARRIVE guidelines recommended for reporting animal research

Laboratory animals	na
Wild animals	We collected adult specimens of the foureye butterflyfish, Chaetodon capistratus for analysis of the gut microbiome. To do so, we followed a fish capture and euthanization protocol approved by the Institutional Animal Care and Use Committee of the Smithsonian Tropical Research Institute (IACUC). Our protocol was optimised to reduce stress and pain on the collected fish. After collection with pole spears, fish were immediately brought to the boat, anesthetized with clove oil and placed on ice.
Field-collected samples	na
Ethics oversight	Institutional Animal Care and Use Committee of the Smithsonian Tropical Research Institute (IACUC); Ministerio de Ambiente Panamá

Note that full information on the approval of the study protocol must also be provided in the manuscript.
